# ADAR1 RNA editing enzyme regulates R-loop formation and genome stability at telomeres in cancer cells

**DOI:** 10.1038/s41467-021-21921-x

**Published:** 2021-03-12

**Authors:** Yusuke Shiromoto, Masayuki Sakurai, Moeko Minakuchi, Kentaro Ariyoshi, Kazuko Nishikura

**Affiliations:** 1 The Wistar Institute, Philadelphia, PA USA; 2 Present Address: Research Institute for Biomedical Sciences, Tokyo University of Science, Chiba, Japan; 3 Present Address: Integrated Center for Science and Humanities, Fukushima Medical University, Fukushima, Japan

**Keywords:** Cancer genomics, RNA editing

## Abstract

ADAR1 is involved in adenosine-to-inosine RNA editing. The cytoplasmic ADAR1p150 edits 3’UTR double-stranded RNAs and thereby suppresses induction of interferons. Loss of this ADAR1p150 function underlies the embryonic lethality of *Adar1* null mice, pathogenesis of the severe autoimmune disease Aicardi-Goutières syndrome, and the resistance developed in cancers to immune checkpoint blockade. In contrast, the biological functions of the nuclear-localized ADAR1p110 remain largely unknown. Here, we report that ADAR1p110 regulates R-loop formation and genome stability at telomeres in cancer cells carrying non-canonical variants of telomeric repeats. ADAR1p110 edits the A-C mismatches within RNA:DNA hybrids formed between canonical and non-canonical variant repeats. Editing of A-C mismatches to I:C matched pairs facilitates resolution of telomeric R-loops by RNase H2. This ADAR1p110-dependent control of telomeric R-loops is required for continued proliferation of telomerase-reactivated cancer cells, revealing the pro-oncogenic nature of ADAR1p110 and identifying ADAR1 as a promising therapeutic target of telomerase positive cancers.

## Abstract (web summary)

One type of RNA editing involves ADAR-mediated conversion of adenosine to inosine. Here the authors show that ADAR1 nuclear isoform p110 regulates R loop formation and genome stability at telomeres in cancer cells.

## Introduction

Adenosine deaminase acting on RNA (ADAR) is the enzyme involved in adenosine-to-inosine RNA editing (A-to-I RNA editing), and three ADAR gene family members (*ADAR1*, *ADAR2*, and *ADAR3*) have been identified in vertebrates^1–5^. ADARs share common domain structures, such as multiple dsRNA-binding domains (dsRBDs) and a separate catalytic domain^6,7^. Both ADAR1 (ADAR, DRADA) and ADAR2 (ADARB1) are catalytically active enzymes, whereas no catalytic activity of ADAR3 (ADARB2) has been shown so far^1–4^. A-to-I editing occurs most frequently in noncoding regions that contain repetitive elements *Alu* and *LINE*^8,9^, and many millions of editing sites have been identified in the human transcriptome of these repetitive sequences^9–11^.

Two ADAR1 isoforms, p150 and p110, are generated by the use of separate promoters and alternate splicing^12^. ADAR1p150 is mostly in the cytoplasm, whereas ADAR1p110 mainly localizes in the nucleus^13^. The cytoplasmic ADAR1p150 regulates the dsRNA-sensing mechanism mediated by melanoma-differentiation-associated protein 5 (MDA5), mitochondrial antiviral signaling protein (MAVS), and interferon signaling (MDA5-MAVS-IFN signaling)^14–16^. The cytoplasmic ADAR1p150 edits 3′-untranslated region (3′-UTR) dsRNAs primarily comprising inverted *Alu* repeats and thereby suppresses activation of MDA5-MAVS-IFN signaling^14–16^. This ADAR1p150 function in the regulation of the MDA5-MAVS-IFN pathway underlies the embryonic lethality of *Adar1*-null mice^17,18^ and also the pathogenesis of Aicardi-Goutières syndrome (AGS; AGS1–7 subgroups known), a severe human autoimmune disease against endogenous nucleic acids^14–16^. Mutations of seven genes, including *RNaseH2A* (AGS4), *RNaseH2B* (AGS2), *RNaseH2C* (AGS3), and *ADAR1* (AGS6), have been identified in association with AGS, and ten AGS6 mutations of *ADAR1* have been reported so far^19^. Finally, this ADAR1p150-mediated suppression of IFN signaling also represses tumor responsiveness to immune checkpoint blockade^20^, revealing the pro-oncogenic ADAR1p150 function. Analysis of The Cancer Genome Atlas database revealed elevated ADAR1 expression and A-to-I editing levels in almost all types of cancers^21,22^, indicating that this pro-oncogenic ADAR1p150 function helps cancer cells suppress inflammatory responses and thus avoid host immunosurveillance^20^. In contrast to the recent advance in the knowledge of ADAR1p150 functions, the biological functions of the nuclear-localized ADAR1p110, other than its involvement in editing of intronic *Alu* dsRNAs^23^, have remained mostly unknown.

Newly transcribed RNA usually dissociates from its template DNA strand immediately after transcription, but occasionally it forms a stable RNA:DNA hybrid, which consequently leaves the sense DNA in a single-stranded form. This structure, called an R-loop, often spans 100–2000 bp and causes abortive transcription and instability of the genome^24,25^. Several mechanisms are known to suppress the formation of R-loops, for example, degradation of RNA strands of RNA:DNA hybrids by RNase H1^26^ and RNase H2^27,28^ and unwinding of RNA:DNA hybrids by helicases such as DExH-box helicase 9 (DHX9)^29^ and senataxin (SETX)^30^. Human diseases including amyotrophic lateral sclerosis type 4, ataxia-ocular apraxia type 2, and AGS are caused by the accumulation of R-loops due to deficiency in one of those suppression mechanisms^31,32^. Telomere end regions consisting of repetitive sequences are important for protection of these regions from recombination and degradation^33,34^. However, telomeric repeat regions are also known to be naturally prone to the formation of R-loops^24,35^, which in turn causes telomere instability and perhaps underlies carcinogenesis of certain cancers^31,36^. The canonical hexameric repeat sequence of the telomeric G-strand (sense strand) is TTAGGG^33,37^. Interestingly, detection of widespread telomeric variant repeats such as TCAGGG and TTGGGG has been reported in cancer cells^38–40^. Mutations/variations (nucleotides) of telomeric canonical repeat DNA sequence TTAGGG (antisense sequence CCCTAA) detected in cancer cells such as TTGGGG (CCCCAA) and TCAGGG (CCCTGA) are emphasized by underlining. Their RNA sequence versions are UUGGGG (CCCCAA) and UCAGGG (CCCUGA). In addition, adenosine residues to be edited by ADAR1 were also emphasized by underlining: TTAGGG (RNA sequence UUAGGG).

In this study, we found an important role for the ADAR1p110 isoform in resolution specifically of the R-loops formed at telomeric repeat regions. ADAR1p110 edits both the RNA and the DNA strands of telomeric repeat RNA:DNA hybrids containing mismatched base pairs formed between canonical and variant repeats. ADAR1p110-mediated editing of A–C-mismatched base pairs, which converts them to I:C-matched base pairs, is required for degradation of the RNA strands of telomeric repeat RNA:DNA hybrids by RNase H2. We found that RNase H2 is incapable of resolving mismatch-containing telomeric RNA:DNA hybrids by itself. This newly found ADAR1p110 role in suppression of telomeric R-loops seems to be essential for the continued proliferation of telomerase-reactivated cancer cells with accumulated variant telomeric repeats, revealing yet another pro-oncogenic function of ADAR1.

## Results

### Depletion of ADAR1 results in telomere DNA damage, telomere abnormalities, and mitotic arrest

**Fig. 1:**
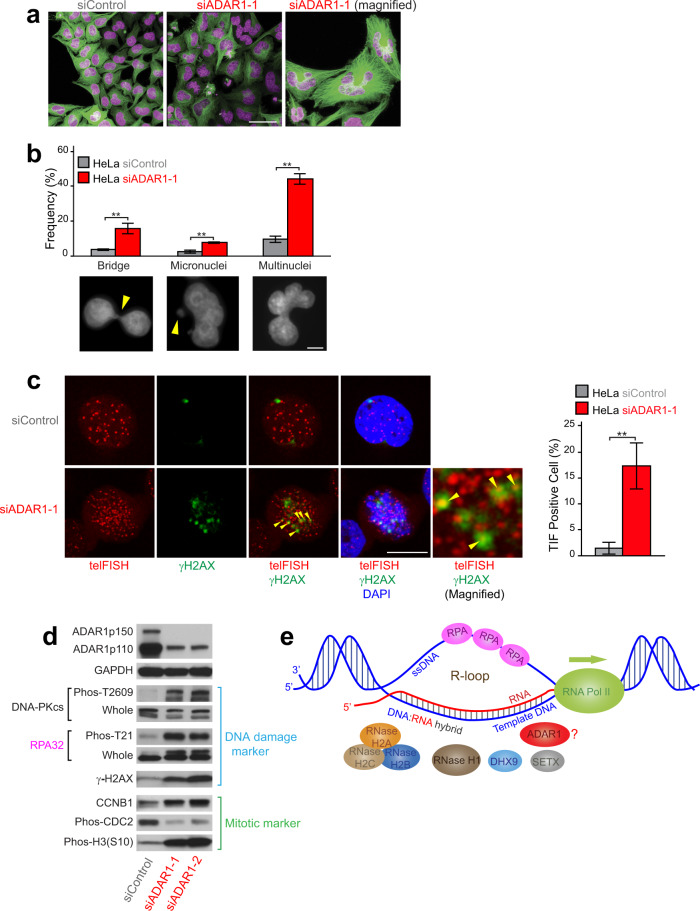
ADAR1 depletion resulted in abnormalities of the nucleus and upregulation of DNA damage pathway and cell cycle marker genes. **a** HeLa cells were first transfected with siControl or siADAR1 (siADAR1-1) for 72 h and then treated with CellLight Tubulin-GFP. Nuclei were visualized by staining of DNA with SiR-DNA reagent. Representative images taken from real-time videos (Supplementary Movies S1 and 2) are presented. Scale bar, 50 μm. **b** The frequency of abnormalities of the nucleus (nucleoplasmic bridge, micronuclei, and multinuclei) was estimated by examining at least 200 individual HeLa cells treated with siControl or siADAR1-1 RNAs. Values are mean ± standard error (*n* = 3, biologically independent samples) with significant differences by two-tailed Student’s *t* test indicated, **P < 0.01. Scale bar, 5 μm. **c** Telomere DNA damages in ADAR1-depleted cells. Telomere FISH and immunostaining for γH2AX revealed significantly increased telomere dysfunction-induced foci (TIF, indicated by yellow arrowheads), suggesting the causative relevance of the telomeric repeat DNA damage to chromosome abnormality detected in ADAR1-depleted HeLa cells. At least 200 individual HeLa cells treated with siControl or siADAR1-1 RNAs were examined. HeLa cells with one or more TIFs were counted as TIF-positive cells. Values are mean ± SD (*n* = 3, biologically independent samples) with significant differences by two-tailed Student’s *t* test indicated, **P < 0.01. Scale bar, 10 μm. **b**, **c** All individual experimental data values and exact *P* values are presented in Source Data file. **d** Western blotting analysis was done using total cell extracts from HeLa cells treated with siControl or two separate siADAR1 (siADAR1-1 and -2) RNAs for 72 h. Protein molecular weight markers are presented in Source Data file. **e** The R-loop structure consisting of an RNA:DNA hybrid formed between the RNA strand newly transcribed by RNA polymerase II and the template DNA strand with the single-stranded antisense DNA bound with ssDNA-binding protein RPA as well as major regulators are schematically shown.

We recently made several observations that indicated the involvement of ADAR1 in the maintenance of telomere stability and mitosis. First, significantly increased telomere abnormality, such as telomere fusions, was detected with *Adar1*-null mouse embryonic fibroblast (MEF) cells derived from *Adar1*-null mouse embryos^18^ (Supplementary Fig. 1a, b). In contrast, no significant telomere abnormality was detected with *Adar2*-null MEF cells (Supplementary Fig. 1c) derived from *Adar2*-null mouse embryos^41^. Chromosome orientation fluorescence in situ hybridization (CO-FISH) analysis revealed significantly increased involvement of leading strands in telomere fusions detected in *Adar1*-null MEF cells (Supplementary Fig. 1d), indicating that ADAR1 is involved in the mechanism that maintains the integrity of the telomere leading strands. Detection of significantly increased *t*elomere dysfunction-*i*nduced *f*oci (TIF) revealed by telomere FISH and γH2AX immunostaining suggested accumulation of DNA damage, if not exclusively, mainly at telomeres in *Adar1*-null MEF cells (Supplementary Fig. 1e), which must be closely related to the increased telomere fusions (Supplementary Fig. 1b, c). Second, time-lapse imaging of HeLa cells undergoing *ADAR1* gene knockdown revealed many aberrantly shaped nuclei (Fig. 1a and Supplementary Fig. 2a) that appeared to be arrested during mitosis, and these aberrant cells eventually died, most likely by apoptosis (Supplementary Movies 1 and 2). Close examination revealed the presence of increased bridged nuclei, micronuclei, and multinuclei in ADAR1-depleted cells (Fig. 1b and Supplementary Fig. 2a). Significantly increased TIFs were detected also in ADAR1-depleted HeLa cells (Fig. 1c). Ectopic expression of ADAR1p110-WT but not ADAR1-E912A, a mutant without catalytic activity^6^ (Supplementary Fig. 2b), suppressed induction of TIFs in ADAR1-depleted cells (Supplementary Fig. 3), demonstrating the importance of ADAR1-mediated A-to-I editing activity in the maintenance of telomere stability. Western blotting analysis revealed significantly upregulated DNA damage markers such as phosphorylated DNA-dependent protein kinase catalytic subunit (DNA-PKcs), γH2AX, and phosphorylated RPA32 in ADAR1-depleted HeLa cells (Fig. 1d). In addition, elevated levels of CCNB1 and histone H3 phosphorylated at serine 10 as well as decreased expression of phosphorylated CDC2 indicated that ADAR1-depleted cells were arrested during mitosis (Fig. 1d). These results together suggest that ADAR1 deficiency causes aberrant mitotic arrest with extensive DNA damage at telomeres.

### Accumulation of R-loops specifically at telomeric repeat regions in ADAR1-depleted cells

**Fig. 2:**
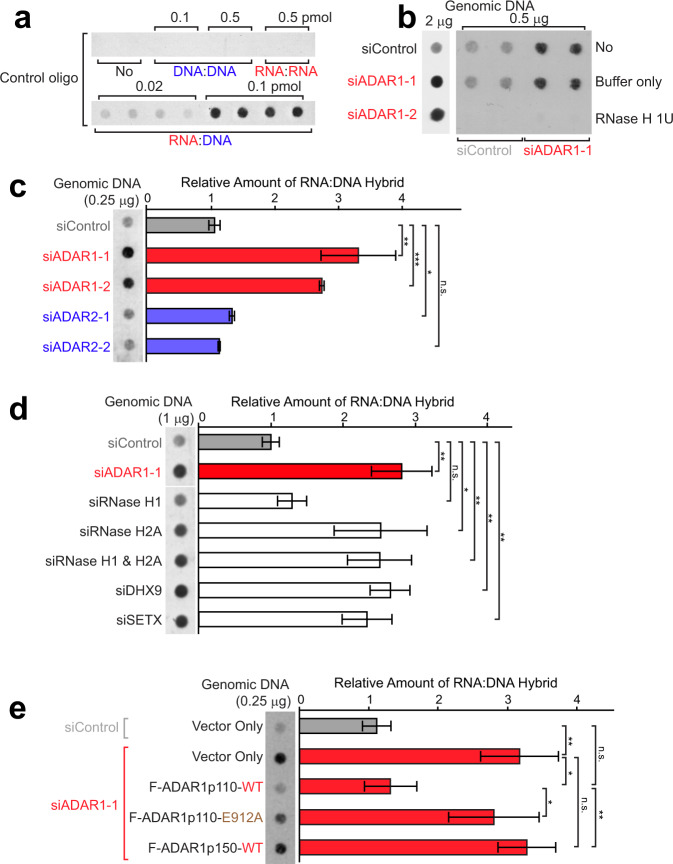
A-to-I editing activity of ADAR1p110, not ADAR1p150 or ADAR2, is required for suppression of R-loops. **a**–**e** Dot blot analysis for RNA:DNA hybrids was conducted using control oligos (**a**) or genomic DNA (**b–e**). **a** The S9.6 antibody recognized specifically RNA:DNA but not DNA:DNA or RNA:RNA oligo duplex controls. **b**, **c** Increased RNA:DNA hybrids were detected only in ADAR1-depleted but not in ADAR2-depleted HeLa cells. **b** The S9.6 antibody signals were abolished by *E. coli*-RNase H treatment, confirming specific detection of RNA:DNA hybrids. **d** Comparison of RNA:DNA hybrid levels between depletion of ADAR1 versus depletion of known R-loop regulators. **e** Increased RNA:DNA hybrid formation resulting from depletion of endogenous ADAR1 was rescued by infection of ADAR1p110-WT (wild type) but not by infection of ADAR1p110-E912A deamination defective mutant or ADAR1p150-WT. **c**–**e** Data are mean ± SD (*n* = 3, biological replicates); significant differences were identified by two-tailed Student’s *t* tests: *P < 0.05, **P < 0.01, ***P < 0.001, n.s., not significant. All individual experimental data values and exact *P* values are presented in Source Data file.

One of the DNA damage markers increased in ADAR1-depleted cells was replication protein A 32 kDa subunit (RPA32 or RPA2) (Fig. 1d). Phosphorylated RPA32 binds to single-stranded DNAs (ssDNAs) and, thus, is an effective marker for R-loops^42^ (Fig. 1e). We reasoned that ADAR1 depletion might result in the accumulation of R-loops, which could cause DNA damage, telomere abnormalities, and mitotic arrest^43–45^. Dot blot analysis using the S9.6 antibody specific to RNA:DNA hybrids^46^ (Fig. 2a) revealed that ADAR1 depletion indeed resulted in significantly increased the formation of RNA:DNA hybrids (Fig. 2b and Supplementary Fig. 2a). Treatment with *Escherichia coli*-RNase H, which digests RNA strands of RNA:DNA hybrids, eliminated dot blot signals, confirming specific detection of RNA:DNA hybrids (Fig. 2b). ADAR2 depletion had almost no effect on the formation of RNA:DNA hybrids (Fig. 2c and Supplementary Fig. 2a), indicating that the R-loop regulatory function is specific to ADAR1.

RNase H1^26^ and H2^28^ degrade RNA strands of RNA:DNA hybrids, while DHX9^29^ and SETX^30^ unwind RNA:DNA hybrids: they all are capable of resolving already formed R-loop structures (Fig. 1e). Depletion of these R-loop regulators, either by single knockdown or combination knockdown of RNase H1 and RNase H2, except for RNase H1 single knockdown (see “Discussion”), resulted in significant accumulation of RNA:DNA hybrids in HeLa cells (Fig. 2d and Supplementary Fig. 2c). Importantly, the amount of RNA:DNA hybrid formed in ADAR1-depleted cells was equivalent to or even more than those formed upon depletion of these known regulators (Fig. 2d). Using various ADAR1-expressing lentivirus systems, we conducted rescue experiments: repression of RNA:DNA hybrids formed in ADAR1-depleted HeLa cells. As expected, ectopic expression of FLAG-tagged ADAR1p110-WT (wild type) efficiently suppressed the formation of RNA:DNA hybrids (Fig. 2e). In contrast, the ADAR1p110-E912A could not rescue despite being expressed at a higher level than that of endogenous ADAR1p110 (Fig. 2e and Supplementary Fig. 2b). Furthermore, ADAR1p150-WT did not suppress the formation of RNA:DNA hybrids (Fig. 2e and Supplementary Fig. 2b). These results demonstrated that A-to-I editing activity of ADAR1p110, but not ADAR1p150, is required for suppression of R-loops.

**Fig. 3:**
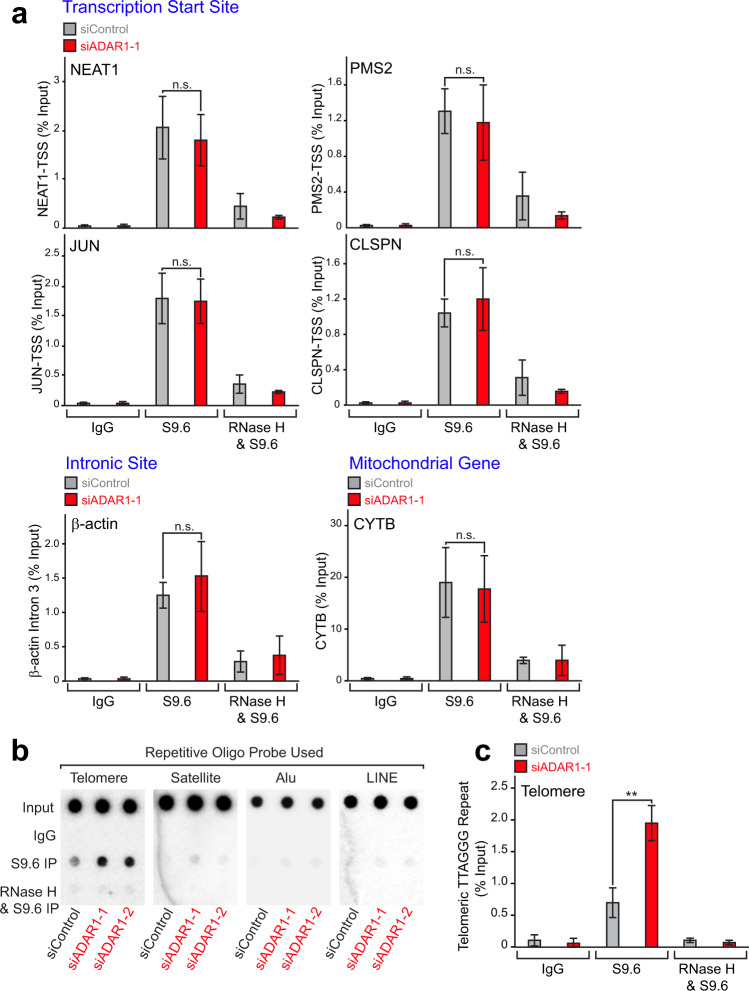
Accumulation of R-loops at telomeres in ADAR1-depleted cells. **a** ADAR1 depletion had no effects on already known sites prone to the formation of R-loops. Six sites were examined by qPCR analysis of DRIP products. PCR primers used are listed in Supplementary Data 1. Data are mean ± SD (*n* = 3, biological replicates); significant differences were identified by two-tailed Student’s *t* tests: n.s., not significant. **b** DRIP products were subjected to genomic DNA dot blot hybridization analysis with a probe containing the G-rich-telomere canonical repeat (TTAGGG), or a probe for α-satellite repeat, *Alu*, or *LINE1* consensus sequence (Supplementary Data 1). ADAR1 depletion resulted in increased the formation of RNA:DNA hybrids specifically at telomeric repeats, which was abolished by *E. coli*-RNase H treatment prior to DRIP. **c** Significance of the increased R-loop formation at telomeric repeats was confirmed by conducting three independent dot blot hybridization analyses of DRIP products. Data are mean ± SD (*n* = 3, biological replicates); significant differences were identified by two-tailed Student’s *t* tests: **P < 0.01, n.s., not significant. **a**, **c** All individual experimental data values and exact *P* values are presented in Source Data file.

We next investigated where in the genome ADAR1p110 controls the formation of R-loops. It has been reported that certain genome loci such as actively transcribed genes and mitochondrial genes are particularly prone to the formation of R-loops^47–49^. Accordingly, we conducted quantitative PCR (qPCR) analysis of DNA:RNA immunoprecipitation (DRIP) products pulled down with the S9.6 antibody. The results revealed that ADAR1 depletion had no effects on the formation of R-loops at the known transcription start sites of *NEAT1*, *JUN*, *PMS2*, and *CLSPN* genes, an intronic site of the *β-actin* gene, and an exonic site of the mitochondrial gene *CYTB*^47–49^ (Fig. 3a). In addition to actively transcribed genes, centromeric and telomeric repeat regions have been reported to be prone to the formation of R-loops^24,25,35^. The repetitive elements of retrotransposons such as *Alu* and *LINE* are known to be the most frequent targets for ADAR1^9,50^. To this end, DRIP products were next tested for dot blot hybridization analysis using four different probes carrying telomeric repeat, α-satellite centromeric repeat, *Alu*, and *LINE1* sequences (Supplementary Fig. 4a, b and Supplementary Data 1). We found that ADAR1 depletion resulted in the accumulation of RNA:DNA hybrids specifically at telomeric repeat regions, but not at α-satellite centromeric repeats nor *Alu* and *LINE1* repeats (Fig. 3b, c), which account for nearly 30% of the human genome^51^. Accumulation of R-loops at telomeres could explain the increased TIFs, various telomere abnormalities observed in *Adar1*-null MEF cells (Supplementary Fig. 1), and mitotic arrest detected in ADAR1-depleted HeLa cells (Fig. 1c, d). We conclude that telomere repeats are, if not exclusive, the major targets of ADAR1.

### ADAR1p110 cannot edit completely complementary telomeric repeat RNA:DNA hybrids

**Fig. 4:**
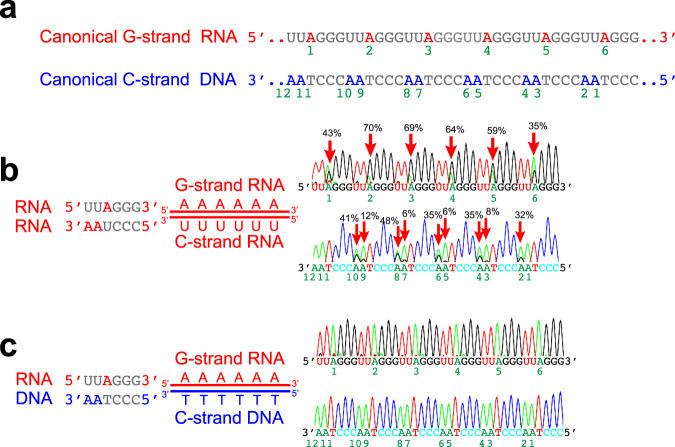
ADAR1p110 cannot edit completely matched RNA:DNA hybrids carrying telomeric repeat sequences. **a** Canonical telomeric repeat sequences of G-strand RNA (red) and C-strand DNA (blue) are shown. Six adenosines of the G-strand RNA and twelve adenosines of the C-strand DNA are indicated by numbers 1–6 and 1–12, respectively. **b** In vitro editing assay for completely matched telomeric repeat dsRNA was conducted using ADAR1p110-WT recombinant protein. **c** In vitro editing assay for completely matched telomeric repeat RNA:DNA hybrids using ADAR1p110-WT recombinant protein. No significant levels of editing for matched RNA:DNA hybrids were detected. **b**, **c** PCR products (RT-PCR-amplified RNA strands and PCR-amplified DNA strands) were subjected to Sanger sequencing. The editing frequency was estimated as the % ratio of the guanosine (black) peak over the sum of guanosine and adenosine (green) peaks of the sequencing chromatograms. Editing frequency estimated for three independent experiments (*n* = 3, technical replicates) is presented in Supplemental information (Supplementary Data 2 and Source Data file).

ADAR1 was originally identified as an A-to-I editing enzyme specific to dsRNAs^52,53^. However, recent studies by Beal and his colleagues^54^ revealed that a catalytic domain fragment of ADAR1 can edit DNA strands of RNA:DNA hybrid substrates in vitro. However, it was not known whether the full-length ADAR1 also had such RNA:DNA editing activity and, if so, whether that RNA:DNA hybrid editing activity is significant in vivo. Interestingly, the adenosine within the hexameric TTAGGG (UUAGGG) sequence of canonical telomeric repeats (Fig. 4a) is the most favored A-to-I editing sites of ADAR1: it prefers 5′ nearest-neighbor U and 3′ nearest-neighbor G^55^. Together with our results (Fig. 3b, c), we reasoned that ADAR1p110 might edit RNA:DNA hybrids carrying telomeric repeat sequences. Editing of A:U or A:T base pairs to I:U or I:T wobble base pairs within telomeric repeat RNA:DNA hybrids would reduce their thermodynamic stability, which in turn would facilitate their dissociation or unwinding by R-loop regulatory helicases such as DHX9 and SETX (Fig. 1e). Accordingly, we prepared telomeric repeat duplex substrates (RNA:RNA and RNA:DNA hybrid) consisting of perfectly complementary sense and antisense strands (Fig. 4b, c) and tested them for in vitro editing with ADAR1p110, the ADAR1 isoform relevant to R-loop regulation (Fig. 2e). As expected, ADAR1p110 very efficiently edited six adenosine residues of the G-strand RNA (Fig. 4b, top), and also, with lower efficiency, C-strand adenosines (Fig. 4b, bottom) of the completely complementary telomeric repeat dsRNA. In contrast, ADAR1p110 editing of the completely matched telomeric repeat RNA:DNA hybrids (Fig. 4c, top and bottom for G-strand RNA and C-strand DNA, respectively) was very inefficient, only at the background level, that is, <5% (Supplementary Data 2), revealing a clear difference in activity toward telomeric repeat dsRNAs versus RNA:DNA hybrids.

### Widespread detection of telomeric variant repeats in ALT and non-ALT cancer cells

**Fig. 5:**
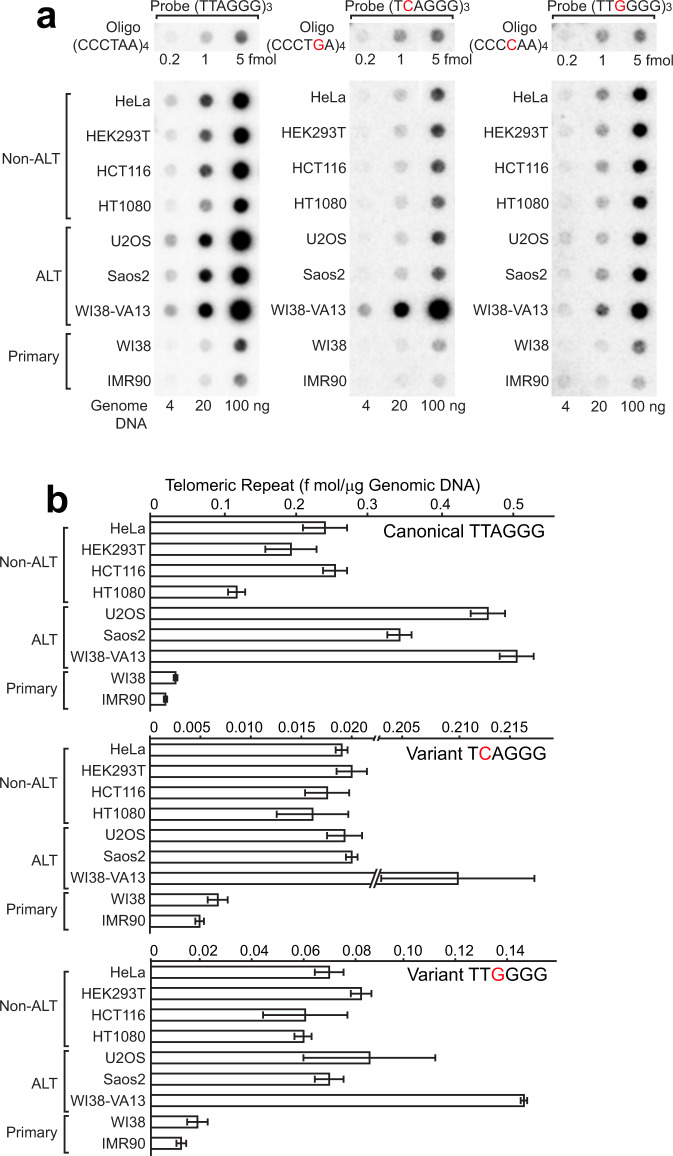
Detection of telomeric variant repeats in ALT-positive and non-ALT cancer cell lines. **a** Dot blot hybridization analysis for genomic DNA samples was conducted using three separate telomeric repeat probes capable of distinguishing a single-nucleotide mismatch (Supplementary Fig. 4a, b). In addition to canonical TTAGGG signals, varying amounts of TCAGGG and TTGGGG variant repeat signals were detected in both ALT and non-ALT cancer cell lines, but not in primary human fibroblast cells. **b** Quantitation of canonical and variant telomeric repeats was done by comparing dot blot signals of genomic DNA and canonical and variant repeat-specific control oligos. Three independent dot blot hybridization analyses were performed. Data are mean ± SD (*n* = 3, biological replicates). All individual experimental data values are presented in Source Data file.

Variant telomeric repeats such as TCAGGG and TTGGGG are detected in the telomeres of cancer cells including HeLa cells. In some cases, the proportion of variant repeats almost equals that of canonical repeats^38,39^. Cancer cells maintain telomere length either by the non-canonical telomere extension mechanism, known as the *a*lternative *l*engthening of *t*elomeres (ALT), in ALT-positive cancer cells (ALT cells) or by reactivating telomerase in ALT-negative cancer cells such as HeLa cells (non-ALT cells)^33,37^. Telomeric variant repeats are amplified by homologous recombination between telomeric repeats in ALT cells, and also by a currently unknown mechanism in non-ALT cells^39^. Using telomeric canonical TTAGGG and variant TCAGGG or TTGGGG repeat-specific probes capable of distinguishing a single-nucleotide mismatch (Supplementary Fig. 4a, b), we detected indeed significant amounts of telomeric variant TCAGGG and TTGGGG repeats in four non-ALT cell lines, including HeLa cells, as well as three ALT cell lines, whereas the amount of telomeric variant repeats was much less in two primary fibroblast cell lines examined (Fig. 5a, b).

### Accumulation of RNA:DNA hybrids containing telomeric variant repeats in ADAR1-depleted cells

**Fig. 6:**
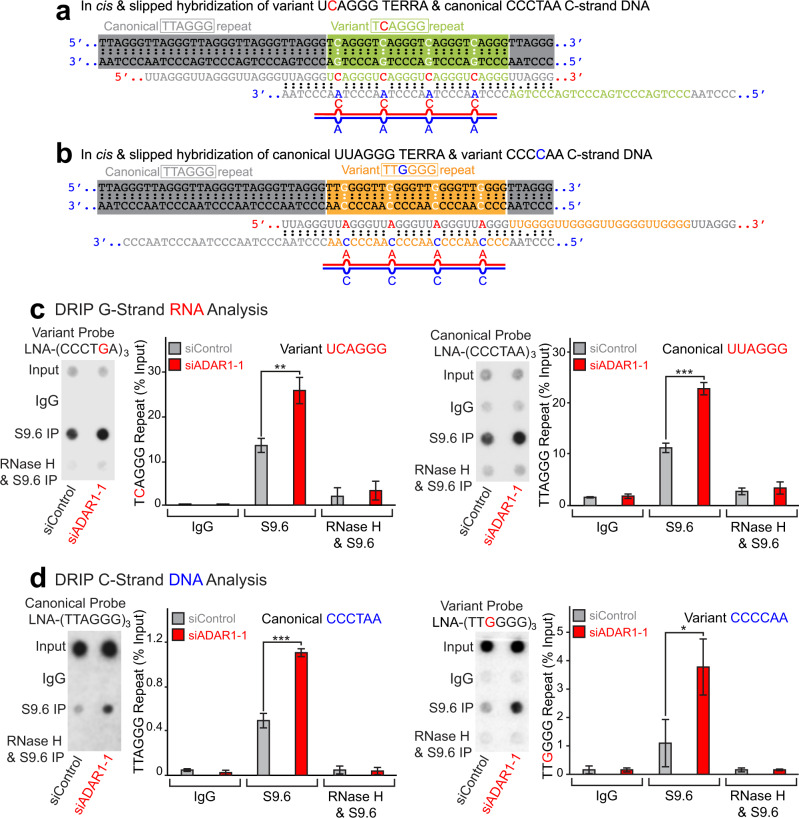
Increased telomeric RNA:DNA hybrids containing variant repeats in ADAR1-depleted cells. Formation of telomeric repeat RNA:DNA hybrids containing A–C mismatches by in *cis* slipped hybridization (**a**, **b**). **a** TERRA RNAs transcribed from the region containing four TCAGGG (green) variant repeats surrounded by TTAGGG (gray) canonical repeats form an RNA:DNA hybrid containing four C–A mismatches by in *cis* slipped hybridization to the C-strand DNA containing canonical TTAGGG (CCCTAA) repeats. **b** TERRA RNAs transcribed from the region containing four TTGGGG (orange) variant repeats surrounded by TTAGGG (gray) canonical repeats form an RNA:DNA hybrid containing four A–C mismatches by in *cis* slipped hybridization to the C-strand DNA containing TTGGGG (CCCCAA) variant repeats. **c**, **d** Detection of increased RNA:DNA hybrids containing TCAGGG and TTGGGG variant repeats in ADAR1-depleted HeLa cells. **c** DRIP products were examined for G-strand RNAs of UCAGGG variant and UUAGGG canonical repeats by dot blot analysis using high-affinity LNA-oligonucleotide probes capable of distinguishing a single-nucleotide mismatch (Supplementary Fig. 4c). **d** Similarly, DRIP products were examined for C-strand DNAs of TTAGGG (CCCTAA) canonical and TTGGGG (CCCCAA) variant repeats using LNA-oligonucleotide probes capable of distinguishing a single-nucleotide mismatch (Supplementary Fig. 4d). **c**, **d** Dot blot signals were abolished by *E. coli*-RNase H treatment prior to DRIP. The significance of the increase in RNA:DNA hybrids containing telomeric canonical and variant repeats (RNA and DNA strands) was confirmed by conducting three independent dot blot hybridization analysis of DRIP products. Data are mean ± SD (*n* = 3, biological replicates); significant differences were identified by two-tailed Student’s *t* tests: *P < 0.05, **P < 0.01, ***P < 0.001. All individual experimental data values and exact *P* values are presented in Source Data file.

Although ADAR1 does edit A:U base pairs of completely matched dsRNAs, A–C-mismatched base pairs present in naturally occurring dsRNAs such as inverted *Alu* dsRNAs are, in fact, the favored ADAR1 target sites^9,50^. Detection of TCAGGG and TTGGGG variant repeats surrounded by TTAGGG canonical repeats within a stretch of telomeric sequence has been reported in HeLa cells^38^. We hypothesized that RNA:DNA hybrids containing A–C-mismatched base pairs could arise in two ways: first, from slipped binding of *te*lomeric *r*epeat-containing *R*N*A*s (TERRAs) derived from a stretch of TCAGGG variant repeats (UCAGGG) to the C-strand of canonical TTAGGG repeats (CCCTAA) (Fig. 6a); second, binding of TERRA RNAs derived from canonical TTAGGG repeats (UUAGGG) to the C-strand of TTGGGG variant repeats (CCCCAA) (Fig. 6b). In particular, TTGGGG variant repeats (both DNA and RNA strands) are prone to the formation of a G-quadruplex structure, which then causes frequent formation of R-loops^56^. G-quadruplex formation of the G-strand TERRAs carrying this particular variant (Fig. 6b, UUGGGG repeats highlighted in orange) is expected to leave its C-strand DNA single stranded, which in turn may cause more frequent slipped hybridization with the canonical repeat G-strand TERRAs (Fig. 6b). Second, there would be an alternative type of pairing, namely in *trans* formation of RNA:DNA hybrids, which has been previously implicated for telomeric repeat sequences^25,57^. Thus, in *trans* hybridization of telomeric repeat RNAs transcribed from one loci either with canonical or variant repeats to C-strand DNAs containing variant or canonical repeats from the other loci, respectively, could also result in telomeric repeat RNA:DNA hybrids containing A–C-mismatched base pairs (Supplementary Fig. 5a, b). G-strand TERRA RNAs containing variant TCAGGG (UCAGGG) and canonical TTAGGG (UUAGGG) repeat sequences are expected to accumulate in C–A (Fig. 6a and Supplementary Fig. 5a) and A–C (Fig. 6b and Supplementary Fig. 5b) mismatch-containing RNA:DNA hybrids, respectively. Similarly, C-strand DNAs containing canonical TTAGGG (CCCTAA) and variant TTGGGG (CCCCAA) repeat sequences are anticipated to be present in C–A (Fig. 6a and Supplementary Fig. 5a) and A–C (Fig. 6b and Supplementary Fig. 5b) mismatch-containing RNA:DNA hybrids, respectively. That is exactly what we observed by dot blot analysis of DRIP products using canonical and variant repeat-specific LNA-oligonucleotide probes (Supplementary Fig. 4c, d): ADAR1 depletion resulted in the accumulation of both RNA and DNA strands of RNA:DNA hybrids consisting of canonical and variant telomere repeats (Fig. 6c, d). These results strongly suggest that ADAR1p110 regulates telomeric RNA:DNA hybrids containing variant repeats and mismatched base pairs.

### ADAR1p110 edits A–C-mismatched base pairs of telomeric RNA:DNA hybrids formed between canonical and variant repeats

**Fig. 7:**
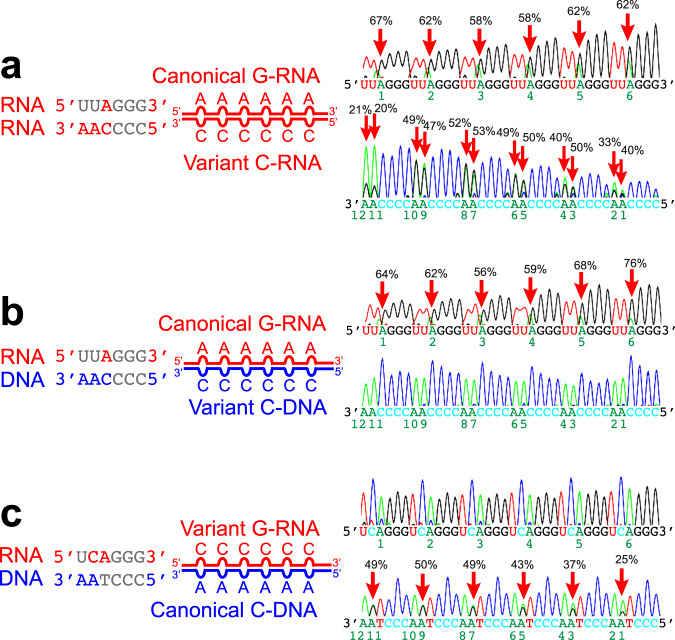
ADAR1p110 edits both RNA and DNA strands of telomeric repeat RNA:DNA hybrids containing A–C mismatches. **a**–**c** In vitro editing assay for telomeric repeat dsRNA (**a**) and RNA:DNA hybrids (**b**, **c**) containing A–C or C–A mismatches was conducted using ADAR1p110-WT recombinant protein. PCR products (RT-PCR-amplified RNA strands and PCR-amplified DNA strands) were subjected to Sanger sequencing. Editing frequency estimated for three independent experiments (*n* = 3, technical replicates) is presented in Supplemental information (Supplementary Data 2 and Source Data file).

To this end, we prepared additional telomeric repeat duplex substrates containing A–C-mismatched base pairs and tested them for in vitro editing with ADAR1p110. ADAR1p110 again edited very efficiently six adenosine residues of telomeric repeat dsRNA containing A–C mismatches, as expected (Fig. 7a, top). In contrast, ADAR1p110 editing of C-strand adenosines of A–C-mismatched dsRNA was less efficient, perhaps due to the fact that two adenosines are not in the most favored UAG sequence context (Fig. 7a, bottom)^55^. Most importantly, ADAR1p110 edited adenosine residues of both the RNA and the DNA strands of RNA:DNA hybrids, provided that they were at mismatched A–C base pairs (Fig. 7b, top and 7c, bottom). The level of editing of the opposite strands of these RNA:DNA hybrids, that is, C-strand DNA and G-strand RNA, respectively, was insignificant (Fig. 7b, bottom and 7c, top). As RNA:DNA hybrid editing activity was lost completely with ADAR1p110-EAA, a dsRNA-binding defective mutant^58^ (Supplementary Fig. 6), binding of ADAR1p110 to RNA:DNA hybrids must be mediated via its dsRBDs, rather than other domains such as Z-DNA-binding domain (Zβ), and is essential for editing of RNA:DNA hybrids. Previous screening has identified ADAR1 as one of many RNA:DNA hybrid-binding proteins, although its biological significance was not addressed^47^.

### Editing of A–C mismatches in telomeric repeat RNA:DNA hybrids facilitates their resolution by RNase H2

**Fig. 8:**
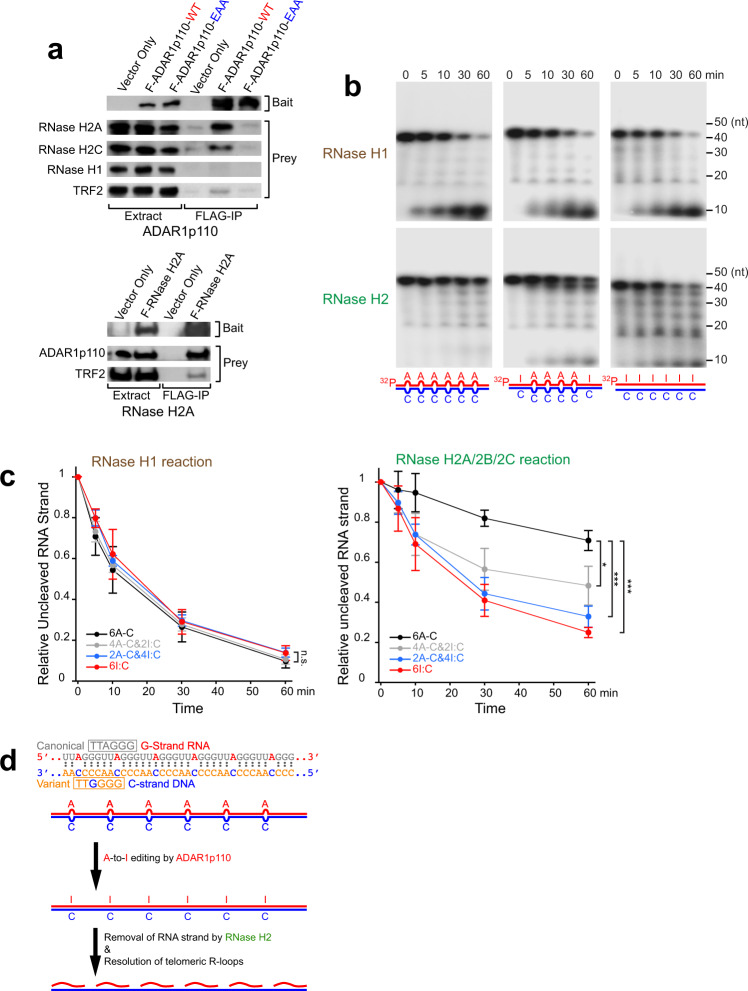
Editing of A–C mismatches in telomeric repeat RNA:DNA hybrids facilitates RNase H2 cleavage of RNA strands. **a** F-ADAR1p110-WT recombinant protein was copurified with endogenous RNase H2 subunits H2A and H2C, but not with RNase H1. No RNase H2 association detected with a dsRNA-binding defective mutant F-ADAR1p110-EAA (upper panels). Similarly, FLAG-RNase H2A pulled down endogenous ADAR1p110 (lower panels). Note that both FLAG-AFAR1p110 and FLAG-RNase H2A pulled down endogenous TRF2, a telomere-binding protein and a member of the Shelterin complex, supporting our hypothesis that ADAR1 and RNase H2 collaborate together to resolve telomeric R-loops. Protein molecular weight markers are presented in Source Data file. **b** In vitro assay for digestion of 5′-^32^P-labeled RNA strands of telomeric repeat RNA:DNA hybrids by RNase H1 or RNase H2A/2B/2C complexes. RNase H2 could not degrade the RNA strands of telomeric repeat RNA:DNA hybrids containing six A–C mismatches, but began digesting RNA strands of RNA:DNA hybrids containing four A–C mismatches. Replacement of all A–C mismatches with I:C-matched base pairs resulted in efficient digestion of RNA strands by RNase H2. RNase H1 degraded RNA strands regardless of the number of A–C mismatches. **c** Time course of digestion of RNA strands of telomeric repeat RNA:DNA hybrids with varying numbers of A–C mismatches by RNase H1 or RNase H2. Data are mean ± SD (*n* = 4, technical replicates); significant differences were identified by two-tailed Student’s *t* tests: *P < 0.05, ***P < 0.001, n.s., not significant. All individual experimental data values and exact *P* values are presented in Supplementary Data 3. **d** A-to-I editing of A–C mismatches to I:C base pairs in RNA:DNA hybrids facilitates digestion of RNA strands by RNase H2.

In order to obtain insight into the mechanism by which ADAR1p110-mediated editing of telomeric repeat RNA:DNA hybrids contribute to resolution of telomeric R-loops, we looked for candidate cofactors of ADAR1p110 required for removal of RNA strands from telomeric RNA:DNA hybrids. We conducted immunoprecipitation experiments using HEK293T cells stably expressing FLAG-ADAR1p110-WT. We found that FLAG-ADAR1p110-WT co-immunoprecipitated with RNase H2A and H2C subunits but not with RNase H1 (Fig. 8a, upper panels), revealing the close association of ADAR1p110 with RNase H2 subunits. Similar experiments with FLAG-ADAR2 revealed no association of ADAR2 with RNase H1 or RNase H2 subunits (Supplementary Fig. 7). The ADAR1p110 interaction with RNase H2 subunits was lost when FLAG-ADAR1p110-EAA dsRNA-binding defective mutant was used as the bait (Fig. 8a, upper panels). Finally, we conducted a reciprocal pull-down experiment using FLAG-tagged RNase H2A as the bait. FLAG-RNase H2A pulled down endogenous ADAR1p110 (Fig. 8a, lower panels). Furthermore, both FLAG-ADAR1p110-WT and FLAG-RNase H2A pulled down TRF2, a telomere-binding protein and a member of the Shelterin complex^33,34^. These results together indicate that ADAR1p110 interacts with RNase H2, but not with RNase H1, on the telomeric RNA:DNA hybrids, and possibly collaborates in dissociating telomeric RNA:DNA hybrids containing mismatched A–C base pairs.

Accordingly, we prepared telomeric repeat RNA:DNA hybrids containing different numbers of A–C-mismatched base pairs as well as I:C-matched RNA:DNA hybrids (mimicking A-to-I-edited hybrids), which were then subjected to in vitro assay using purified human recombinant RNase H1 and RNase H2A/2B/2C complex proteins. To our surprise, RNase H1 digested RNA strands of telomeric repeat RNA:DNA hybrids regardless of the number of A–C-mismatched base pairs (Fig. 8b, upper panels and 8c, left). In contrast, we found that digestion by RNase H2 is very sensitive to the presence of A–C-mismatched base pairs. The RNA strand of the RNA:DNA hybrids containing six A–C-mismatched base pairs was almost completely resistant to digestion by RNase H2 (Fig. 8b, lower panels and 8c, right). As A–C-mismatched base pairs are replaced with matched I:C base pairs, the RNA:DNA hybrids became more permissive to RNase H2-mediated digestion (Fig. 8c, right): more efficient digestion for hybrids with more I:C-matched base pairs (Fig. 8d and Supplementary Fig. 8).

### ADAR1 depletion leads to the accumulation of RNA:DNA hybrids only in non-ALT cancer cells

**Fig. 9:**
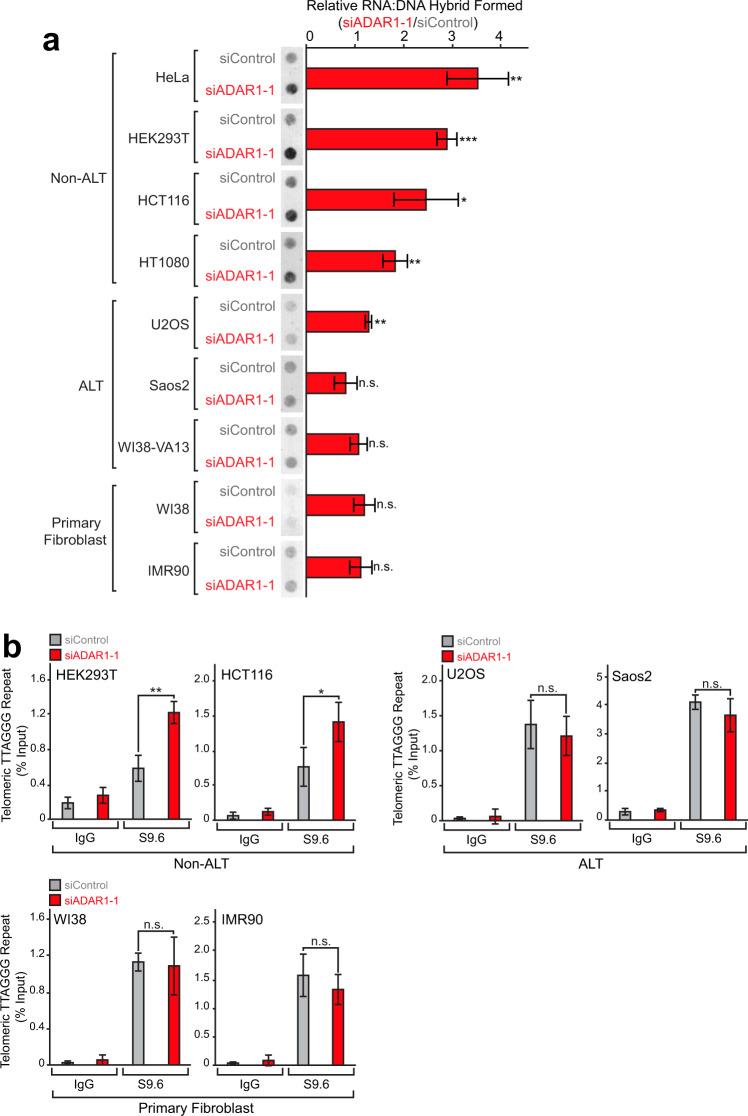
ADAR1 regulates accumulation of telomeric R-loops only in non-ALT cells. **a** Detection of increased RNA:DNA hybrids only in non-ALT cells. Genomic DNA samples (0.25 µg) collected from various cells treated with siControl or siADAR1-1 RNAs for 72 h were examined by dot blot assay using the S9.6 antibody. Data are mean ± SD (*n* = 3, biological replicates); significant differences were identified by two-tailed Student’s *t* tests: *P < 0.05, **P < 0.01, ***P < 0.001, n.s., not significant. **b** DRIP products from select cell lines were further subjected to dot blot analysis for the C-strand telomeric repeat DNA using the canonical telomeric repeat (TTAGGG) specific probe (Supplementary Fig. 4a). Telomeric repeat RNA:DNA hybrids were detected only in HEK293T and HCT116 non-ALT cancer cell lines. Data are mean ± SD (*n* = 3, biological replicates); significant differences were identified by two-tailed Student’s *t* tests: *P < 0.05, **P < 0.01, n.s., not significant. **a**, **b** All individual experimental data values and exact *P* values are presented in Source Data file.

Our FLAG-ADAR1p110 immunoprecipitation experiments revealed the association of ADAR1p110 with RNase H2 subunits, but not with RNase H1 (Fig. 8a). Efficient resolution of telomeric RNA:DNA hybrids by RNase H1 has been reported specifically with ALT activity positive cancer cell lines (ALT cells)^45^. Interestingly, the same study also reported that RNase H1 had no or very little effects on resolution of telomeric RNA:DNA hybrids in non-ALT cell lines^45^, indicating different mechanisms used for regulation of telomeric R-loops in ALT versus non-ALT cancer cells. Accordingly, we investigated the effects of ADAR1 depletion on accumulation of RNA:DNA hybrids in both ALT and non-ALT cell lines as well as primary fibroblast cells. ADAR1 depletion resulted in increased RNA:DNA hybrids in all non-ALT cell lines examined, but had almost no effects in ALT cell lines nor in primary fibroblast cells (Fig. 9a and Supplementary Fig. 2d). Furthermore, dot blot analysis of DRIP products using a telomeric repeat probe revealed that ADAR1 depletion resulted in the formation of telomeric repeat RNA:DNA hybrids specifically in non-ALT cancer cell lines, for example, HeLa (Fig. 3c), HEK293T, and HCT116 (Fig. 9b and Supplementary Fig. 9). The reported association of RNase H1 with the strong telomeric repeat R-loops formed only in ALT cells^45^, perhaps highlights the capability of RNase H1 to resolve telomeric RNA:DNA hybrids even in the presence of mismatched base pairs (Fig. 8b, c).

**Fig. 10:**
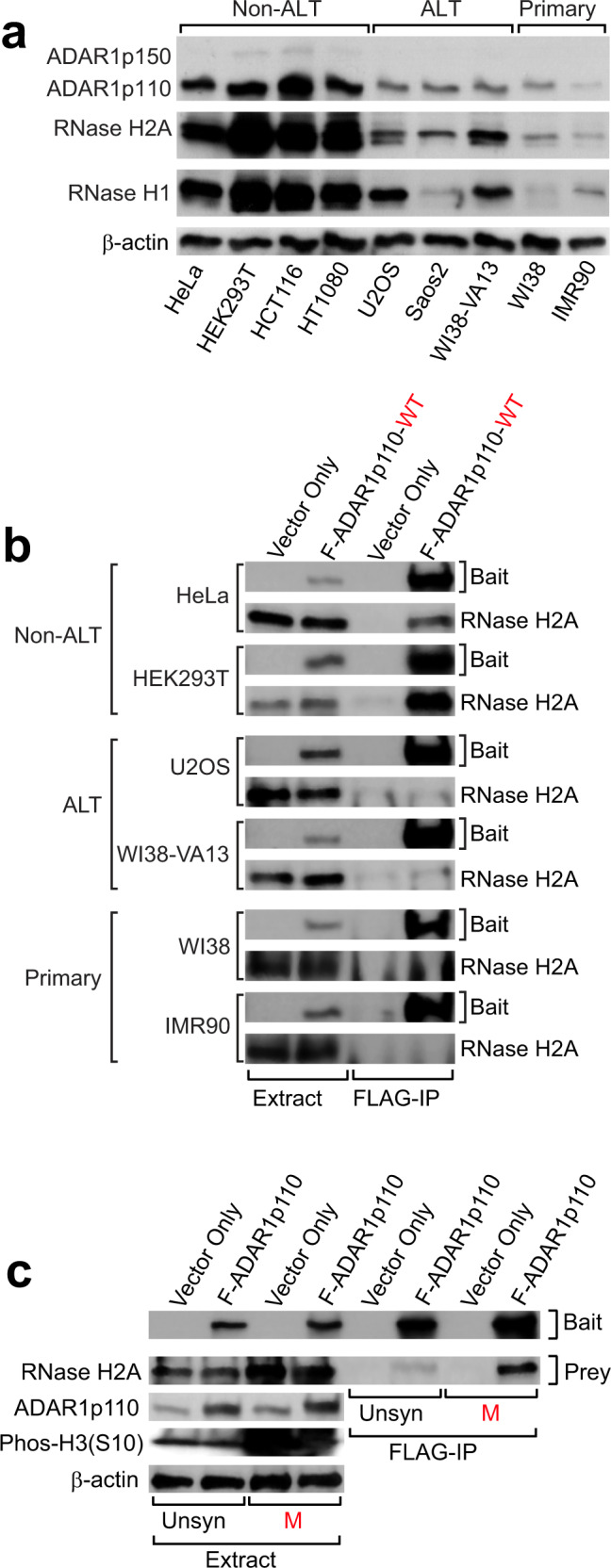
M-phase-specific interaction of ADAR1p110 and RNase H2 in non-ALT cancer cells. **a** Elevated ADAR1 and RNase H2A expression levels detected in non-ALT cells. Western blotting analysis was performed using total cell extract proteins and specific antibodies (Supplementary Data 4). **b** Interaction between ADAR1p110 and RNase H2 detected only in non-ALT cancer cells. FLAG-ADAR1p110-WT recombinant protein was copurified with endogenous RNase H2 subunit H2A only from non-ALT cell lines. **c** Elevated RNase H2A expression levels and also increased interaction of ADAR1p110 with RNase H2A detected specifically at M phase. FLAG-ADAR1p110-WT pull-down experiments were conducted using HEK293T cells synchronized at M phase using the thymidine-nocodazole double block system. **a**–**c** Protein molecular weight markers are presented in Source Data file.

We found that ADAR1p110 and RNase H2A expression levels are much higher in non-ALT cancer cells as compared to those in ALT cancer cells and primary fibroblast cells (Fig. 10a). Furthermore, the interaction of ADAR1p110 with RNase H2A was detected only in non-ALT cancer cells, but not in ALT cancer cells and primary fibroblast cells (Fig. 10b). Interestingly, ADAR1 depletion resulted in the upregulation of M-phase-specific marker genes (Fig. 1d), perhaps indicating an M-phase-specific ADAR1p110 function in suppression of telomeric R-loops. Accordingly, we analyzed the interaction of ADAR1p110 and RNase H2 by F-ADAR1p110 co-immunoprecipitation (co-IP) experiments in cells synchronized to the M phase. First, we noticed that RNase H2A expression levels increased significantly, >2-fold, at the M phase (Fig. 10c, compare unsynchronized and M-phase extract lanes), while endogenous ADARp110 levels unchanged (Fig. 10c, compare unsynchronized and M-phase extract vector-only lanes). Most importantly, we found that the ADAR1p110–RNase H2A interaction increased >3-fold in M-phase synchronized cells as compared to unsynchronized cells (Fig. 10c, FLAG-IP lanes). These results perhaps indicate a special need in non-ALT cancer cells for two-step removal of RNA strands of telomeric RNA:DNA hybrids accumulated specifically around late G2 to M phase: first correction of mismatched base pairs by the nuclear ADAR1p110 and then for degradation of RNA strands by RNase H2 (Fig. 8d and Supplementary Fig. 8). Taken together, our results indicate a differential role played by RNase H1 and RNase H2 in resolution of R-loops accumulated in ALT and non-ALT cancer cells, respectively, due to their different activity in degrading mismatch-containing telomeric RNA:DNA hybrids (Supplementary Fig. 10).

## Discussion

Several regulators of telomeric R-loops such as 5′ to 3′ exonuclease, Rat1p^59^, flap endonuclease 1^60^, and RNase H1 and H2^45,59,61^ have been reported. In this study, we identified ADAR1p110 as a major regulator of telomeric R-loops specifically in cancer cells. Variant telomeric repeats such as TCAGGG and TTGGGG repeats are widespread in both ALT and non-ALT cancer cell lines^38,39^ (Fig. 5). Because of these variant repeats, cancer cells encounter a unique problem in solving telomeric RNA:DNA hybrids containing mismatched base pairs. Hybridization of TERRA molecules carrying the G-strand UCAGGG variant sequences to the C-strand DNA carrying the canonical CCCTAA (antisense of TTAGGG) repeats (Fig. 6a and Supplementary Fig. 5a) or hybridization of TERRA molecules carrying the G-strand canonical UUAGGG sequence to the C-strand DNA carrying the CCCCAA (antisense of TTGGGG) variant sequence (Fig. 6b and Supplementary Fig. 5b), either by slipped hybridization or in *trans* hybridization, would result in the formation of telomeric repeat RNA:DNA hybrids containing C–A or A–C mismatches, respectively. The possibility of R-loop formation induced by mismatches between nascent RNA and DNA sequences has been previously pointed out^62^.

We found that ADAR1p110 could edit efficiently A–C-mismatched adenosines of both RNA and DNA strands within telomeric RNA:DNA hybrids and convert them to I:C-matched Watson–Crick base pairs. Interestingly, telomeric variant repeats have been reported to expand during ALT-mediated inter- and/or intra-telomeric recombination in ALT cells, and by a currently unknown mechanism in non-ALT cells^38,39^. An interesting possibility arises upon A-to-I editing of these mismatched A–C base pairs to I:C-matched base pairs: replication of A-to-I-edited C-strand DNA could generate more variant telomeric TCAGGG repeats (Supplementary Fig. 8, bottom), perhaps explaining the reported expansion of this variant repeat in cancer cells^38,39^. Most importantly, we found that A-to-I editing of A–C mismatches within RNA:DNA hybrids is critical for efficient digestion of RNA strands by RNase H2, and consequent resolution of telomeric R-loops (Fig. 8d and Supplementary Fig. 8), because RNase H2, unlike RNase H1, is incapable of digesting RNA strands of mismatch-containing RNA:DNA hybrids. Our findings on association of ADAR1p110 with RNase H2, but not with RNase H1 in living cells (Fig. 8a), together with elevated expression of RNase H2A subunit (Fig. 10a), especially at the M phase (Fig. 10c), further confirms collaboration between ADAR1p110 and RNase H2 in resolving specifically telomeric RNA:DNA hybrids containing A–C mismatches, perhaps at late G2 to M phase, in non-ALT cancer cells (Supplementary Fig. 10).

We currently do not know the exact reason why a specific requirement of ADAR1p110 for suppression of telomeric R-loops is restricted to non-ALT cells (Fig. 9a, b and Supplementary Fig. 10), as widespread presence of telomeric variant repeats has been detected in both ALT and non-ALT cancer cells (Fig. 5). The cell cycle-dependent function of RNase H2, specifically at G2–M phase, in resolution of R-loops, has been reported also in yeast^63^. In contrast, RNase H1 has been reported to act through the entire cell cycle in yeast^63^. We confirmed elevated expression of RNase H2 and its interaction with ADAR1p110 specifically at the M phase in non-ALT cancer cells (Fig. 10c). Most interestingly, an association of RNase H1 with telomeric RNA:DNA hybrids at strong R-loop-forming loci has been reported only in ALT cancer cells with overly elevated TERRA expression levels^45^ (Supplementary Fig. 10, left). Association of RNase H1 to telomeric R-loops may also be further facilitated by more frequent binding of RPA32 to the single-stranded G-strand DNA specifically in ALT cells^64^. RPA32 has been shown to enhance the association of RNase H1 and R-loops^42^ (Supplementary Fig. 10, left). Knockdown of RNase H1 in HeLa cells (non-ALT cells) indeed did not lead to a significant increase of R-loops (Fig. 2d), in agreement with the previous report: RNase H1-dependent resolution of telomeric R-loops occurs only in ALT cells^45^ (Supplementary Fig. 10, left). TERRA expression levels seem to be another factor that differentiates non-ALT cells from ALT cells. In non-ALT HeLa cells, expression of TERRA is regulated in a cell cycle-dependent manner: lowest in S and early G2, but increasing toward the end of G2 and M phase^65^. Furthermore, TERRA expression levels remain low in non-ALT cells such as HeLa and HEK293T, and thus unable to support strong R-loop-forming loci and recruit RNase H1^45^ (Supplementary Fig. 10, right). Instead, RNase H2 together with ADAR1p110 is recruited to such not so strong R-loop-forming loci during G2–M phase, and RNase H2 and ADAR1p110 cooperatively resolve telomeric repeat RNA:DNA hybrids containing mismatched base pairs (Supplementary Fig. 10, right). Clearly, many issues remain to be resolved with regard to why and how two mechanisms are differentially employed for resolution of telomeric R-loops in ALT and non-ALT cancer cells.

Telomere abnormalities such as telomere losses and telomere leading-strand-mediated fusions, most likely caused by unresolved telomeric R-loops, were detected in ADAR1-depleted cells (Supplementary Fig. 1). Although we have no direct evidence to specify the exact timing, telomeric RNA:DNA hybrids may form and accumulate from the late S phase through M phase in ADAR1-depleted HeLa cells. Unresolved telomeric RNA:DNA hybrids as well as prolonged exposure of single-stranded G-strands most likely lead to double-stranded DNA breaks and eventually to telomere losses and fusions. Apparently, the telomerase activated in non-ALT cells does not mend the shortened telomere ends. Non-homologous end joining and DNA-PKcs participate in telomere end-capping, exclusively at telomeres generated by leading-strand synthesis in non-ALT cancer cells^66,67^. This end-capping process does not seem to function efficiently in ADAR1-depleted cells. It has been reported that unresolved and persistent R-loops interfere with the DNA double-strand break (DSB) repair mechanism^68^. Although factors involved in DSB  repair, such as DNA-PKcs and γH2AX, are activated in ADAR1-depleted cells (Fig. 1d), the DSB repair mechanism may be hindered due to the presence of unresolved RNA:DNA hybrids, which could also contribute to the telomere abnormalities detected. Additional and specific studies will be required to address these issues.

Recent elegant studies by Nicholas Haining and his colleagues^20^ revealed the possibility that ADAR1 inhibitors could restore MDA5-MAVS-IFN signaling and inflammatory responses in tumors and resurrect their response to therapy utilizing immune checkpoint blockade. However, our studies presented here suggest another possibility: elimination of ADAR1 and/or suppression of its A-to-I editing activity would lead to the accumulation of telomeric repeat R-loops and consequent genome instability and apoptosis particularly in non-ALT and telomerase-positive cancers, which are, in fact, 70–80% of all types of cancers^45^. We predict that ADAR1 inhibitors would be very effective therapeutics for cancer treatment because they will interfere with two completely different pro-oncogenic ADAR1 functions: suppression of MDA5-MAVS-IFN signaling by the cytoplasmic ADAR1p150 and maintenance of telomere stability in telomerase-reactivated cancer cells by the nuclear ADAR1p110.

## Methods

### Cell lines and cell culture reagents

HeLa human ovarian carcinoma (ATCC CCL-2), HEK293T human embryonic kidney (ATCC CRL-11268), HCT116 human colon carcinoma (ATCC CCL-247), HT1080 human fibrosarcoma (ATCC CCL-121), U2OS human osteosarcoma (ATCC HTB-96), WI38-VA13 human virus-transformed fibroblasts (ATCC CCL-75.1), Saos2 human osteosarcoma (ATCC HTB-85), WI38 lung fibroblast (ATCC CCL-75), and IMR90 lung fibroblast cells (ATCC CCL-186) were used in this study. *Adar1*^−/−^ MEF cells and isogenic control cells were established from *Adar1*^−/−^ mice^18^. *Adar2*^−/−^ MEF cells and isogenic control cells were established from *Adar2*^−/−^ mice^41^. HEK293T cells expressing FLAG-ADAR1p110-WT, FLAG-ADAR1p110-EAA, FLAG-ADAR2-WT, or FLAG-ADAR2-EAA were established by co-transfection of various p3XFLAG-CMV-10 plasmids (Sigma) with a puromycin resistance plasmid pPUR (Clontech)^69^. These cell lines were free of mycoplasma contamination.

HeLa, HEK293T, HCT116, HT1080, Saos2, IMR90, *Adar1*^−/−^ MEF, *Adar2*^−/−^ MEF cells, and isogenic control MEF cells were cultured in Dulbecco’s modified Eagle’s medium (DMEM) supplemented with 10% fetal bovine serum (Gemini), penicillin (100 U/ml), and streptomycin (100 μg/ml) at 37 °C in a humidified atmosphere with 5% CO_2_. U2OS, WI38-VA13, and WI38 cells were cultured in DMEM/F12 supplemented with 10% fetal bovine serum, penicillin (100 U/ml), and streptomycin (100 μg/ml).

HEK293T cells expressing FLAG-ADAR1p110-WT were treated with thymidine and nocodazole to synchronize in the M phase. The cells were cultured in T175 flask and treated with 2.5 mM of thymidine (Sigma) for 24 h. To release from the thymidine block, the cells were washed twice with phosphate-buffered saline (PBS) and culture medium. After incubation with a fresh medium for 3 h, the cells were treated with 0.1 μg/ml of nocodazole (Sigma) for 12 h.

### Plasmid construction

An *Nhe*I restriction site was added to the multi-cloning site (MCS) of CSII-EF-MCS-IRES-puromycin-resistant gene (puro) by inserting the new MCS site oligonucleotide into *Not*I–*Bam*HI-digested CSII-EF-MCS-IRES-puro vector^70,71^. CSII-EF-FLAG-ADAR1p110-WT-IRES-puro, CSII-EF-FLAG-ADAR1p110-E912A-IRES-puro, or CSII-EF-FLAG-ADAR1p150-WT-IRES-puro used for protein overexpression in human cells was prepared by PCR cloning using p3XFLAG-CMV10-ADAR1p110-WT, p3XFLAG-CMV10-ADAR1p110-E912A, or p3XFLAG-CMV10-ADAR1p150-WT, respectively^69^. The FLAG-ADAR1p110 PCR products were amplified using primers CSII-FLAG-p110-F and CSII-FLAG-p110-R. The PCR products were digested with *Not*I and *Bam*HI and then inserted into CSII-EF-MCS-IRES-puro. The FLAG-ADAR1p150 PCR products were amplified using primers CSII-p150-F and CSII-p150-R. The PCR products were digested with *Not*I and *Nhe*I and then inserted into CSII-EF-MCS-IRES-puro. CSII-EF-FLAG-RNaseH2A was prepared by PCR cloning using pcDNA3.1-RNaseH2A^72^ The FLAG-RNaseH2A PCR products were amplified using primers CSII-FLAG-RNaseH2A-F and CSII-RNaseH2A-R. The PCR products were digested with *Not*I and *Bam*HI and then inserted into CSII-EF-MCS-IRES-puro. CSII lentivirus vector was a kind gift from Hiroyuki Miyoshi and Toru Nakano.

pET28-FLAG-RNaseH2A used for recombinant protein purification was prepared by PCR cloning using a pET28-His-RNaseH2A plasmid. The PCR products were amplified using primers pET28-FLAG-RNaseH2A-F and FLAG-RNaseH2A-R, digested with *Xba*I and *Xho*I, and inserted into pET28 vector^73^. pET28-His-RNASEH2A and pET15-His-RNaseH2B/2C were kind gifts from Marcin Nowotny. Oligo DNAs used for plasmid construction are listed in Supplementary Data 1.

### Gene knockdown

Gene knockdown experiments were done by RNA interference methods using Lipofectamine RNAiMax (Life Technologies) or HiperFect at a final short interfering RNA (siRNA) concentration of 1, 2, or 5 nM. All siRNAs used in this study are listed in Supplementary Data 1.

### Lentivirus infection

HEK293FT cells (5–6 × 10^6^) incubated in a 10 cm dish for a confluency of 80% were transfected with the following three plasmids using Lipofectamine 3000: 17 µg of CSII-EF-FLAG-ADAR1 or CSII-EF-FLAG-RNaseH2A plasmid, 10 µg of pCAG-HIVgp (GAG-POL DNA), and 10 µg of the vesicular stomatitis virus G (VSV-G) envelope plasmid pCMV-VSV-G. After 48 h incubation, the cell culture supernatant was filtered using a 0.45 μm filter and concentrated by Lenti-X concentrator (Clontech). The lentiviral pellet was resuspended in 2 ml of fresh culture medium containing 8 µg/ml of polybrene (Sigma) and added to 1 × 10^5^ cells cultured in a 6-well plate. Infected cells were then incubated with puromycin (1 μg/ml) for 48 h post infection for antibiotic selection. The extent of infection of CSII-EF-FLAG-ADAR1 or CSII-EF-FLAG-RNaseH2A was evaluated by western blotting analysis and immunostaining with anti-FLAG M2 antibody. ADAR1 rescue experiments required exogenous FLAG-ADAR1 expression in every cell and were carried out using early passage cells (≤passage 6).

### Immunofluorescence staining

Transfection of siRNAs (siADAR1-1) into HeLa cells at 1 nM concentration was carried out as described above. After incubation for 24 h, the culture medium was replaced with a fresh medium containing CellLight Tubulin-GFP and BacMam 2.0 (Thermo Fisher Scientific). Nuclei were stained with SiR-DNA reagent (Cytoskeleton) at 0.25 μM for 6 h. Cells were cultured on Ibidi μDish 3.5 cm. After 72 h, cells were fixed with 4% paraformaldehyde and soaked in Dulbecco’s PBS. Microscopic images were obtained by using a Leica TCS SP5 DMI6000 CS Confocal Microscope and LAS X software (Leica), equipped with ultraviolet 405 diode, Argon, DPS3561, and HeNe594 lasers. Fluorescent images were captured with a 40× lens with a 512 × 512 frame. For multicolor experiments, the following wavelength settings were used: Tubulin-GFP (Ex 488 nm/Em 498–630 nm) and SiR-DNA reagent (Ex 647 nm/Em 657–800 nm). Nuclear morphological analysis was performed using 4′,6-diamidino-2-phenylindole (DAPI)-stained HeLa cells.

### Immunoblot analysis

Cell lysates were prepared in Laemmli buffer containing benzonase nuclease (Sigma), complete EDTA-free proteinase inhibitor cocktail (Roche), and PhosStop phosphatase inhibitor cocktail (Roche) and size-fractionated by 4–20% sodium dodecyl sulfate-polyacrylamide gel electrophoresis. Proteins were blotted to Immobilon-P nylon membrane (Millipore). Membranes were blocked with 10% Blocker BSA (bovine serum albumin) buffer (Thermo Fisher Scientific) and incubated with the primary antibodies overnight at 4 °C. After incubation with each appropriate secondary antibody, bands were detected with ECL (GE Healthcare) using X-ray films. Antibodies were diluted in 10% Blocker BSA buffer (Thermo Fisher Scientific). Antibodies used in this study are listed in Supplementary Data 4.

### Dot blot analysis of genomic DNA

Cells were treated with siRNA for 72 h in a 10 cm dish, detached from the dish surface with TrypLE Express Enzyme, and harvested by centrifugation. After PBS wash, genomic DNA was purified using QIAGEN Blood & Cell Culture DNA Midi Kit. Briefly, the cell pellet was resuspended in buffer C1. After repeated buffer C1 wash and removal of the cell debris, the nuclear pellet was resuspended in buffer G2 (without RNase A) and treated with 2 mg of proteinase K at 50 °C for 60 min. The nuclear fraction was applied to a buffer QBT-treated QIAGEN Genomic-tip and washed twice with buffer QC. Genomic DNA was eluted with buffer QF and precipitated with 2-propanol. The DNA pellet was washed twice with 80% ethanol and air-dried. Genomic DNA was dissolved in TE buffer (10 mM Tris-HCl pH 8.0, 1 mM EDTA) and incubated overnight at 4 °C.

Genomic DNA was diluted in 100 μl of 6× saline sodium citrate (SSC) and spotted onto a Hybond N^+^ (GE Healthcare) using a Bio-Dot Apparatus (#1706545, Bio-Rad). The membrane was cross-linked with ultraviolet (UV) (0.24 J) and blocked with 5% non-fat dry milk (LabScientific) in PBS with 0.1% Tween-20 (PBST) for 1 h and then with SuperBlock buffer (Thermo Fisher Scientific) for 1 h at room temperature. The membrane was incubated with S9.6 antibody (Sigma) at 0.1 μg/ml in SuperBlock buffer overnight at 4 °C. After washing three times with PBST, the membrane was incubated with horseradish peroxidase-conjugated donkey anti-mouse IgG secondary antibody (Jackson Immuno Research) (0.04 μg/ml) at 0.1 μg/ml in SuperBlock buffer for 1 h at room temperature. After washing four times with PBST, dot signals were detected with ECL (GE Healthcare) using X-ray films. For the treatment with RNase H, 1 μg of genomic DNA was preincubated with 2 U of *E. coli*-RNase H (NEB) for 2 h at 37 °C. DNA:DNA, RNA:RNA, or RNA:DNA oligo duplex controls were annealed in buffer containing 10 mM Tris-HCl (pH 7.6) and 50 mM NaCl at 80 °C for 5 min, followed by slow cooling to room temperature. Oligonucleotides were used as controls are listed in Supplementary Data 1.

### DNA:RNA hybrid immunoprecipitation

Fifty micrograms of genomic DNA prepared as described above was diluted in 250 μl of sonication buffer (10 mM Tris-HCl pH 8.5, 300 mM NaCl) and sonicated using Bioruptor (Diagenode) (20 cycles at high power, 30 s ON/60 s OFF) or Sonicator W-220 (Heat Systems Ultrasonics) (20 cycles at Lv3.5, 10 s ON/40 s OFF). During sonication, samples were kept cold very carefully. Sonicated genomic DNA was mixed with 0.02 pmol spike RNA:DNA oligonucleotide duplexes. A fraction of the genomic DNA sample (90%, 225 μl) was used for immunoprecipitation with the S9.6 antibody (Sigma), and the remaining fraction (10%, 25 μl) was used as the input control. Protein A beads (Dynabeads Protein A, Invitrogen) (100 μl) were blocked with 0.5% BSA in PBS containing 5 mM EDTA overnight at 4 °C. After washing twice with DRIP buffer (50 mM Tris-HCl pH 7.4, 150 mM NaCl, 5 mM EDTA, 1% NP-40, 0.1% sodium deoxycholate), 20 μg of S9.6 antibody (Sigma) or control mouse IgG (sc-2025, Santa Cruz) were applied to the blocked Dynabeads in DRIP buffer overnight at 4 °C. After washing twice with DRIP buffer, the beads were resuspended in 100 μl of DRIP buffer containing 500 U of RNasin Plus inhibitor (Promega). Sonicated DNA was diluted to half concentration in a buffer containing 100 mM Tris-HCl pH 7.4, 10 mM EDTA, 2% NP-40, and 0.2% sodium deoxycholate. Then, 100 μl of the beads were added to DNA solution and incubated overnight at 4 °C with rotation. The beads were washed by the following steps: (1) twice with DRIP buffer; (2) twice with DRIP high buffer (50 mM Tris-HCl, pH 7.4, 500 mM NaCl, 5 mM EDTA, 1% NP-40, 0.1% sodium deoxycholate); (3) twice with DRIP Li buffer (50 mM Tris-HCl, pH 8.0, 250 mM LiCl, 1 mM EDTA, 0.5% NP-40, 0.5% sodium deoxycholate); (4) once with DRIP TE NaCl+ buffer (100 mM Tris-HCl pH8.0, 10 mM EDTA, 50 mM NaCl); and (5) once with DRIP TE buffer (100 mM Tris-HCl pH8.0, 10 mM EDTA). After removal of DRIP TE buffer using a magnetic stand, RNA:DNA hybrids were eluted from the beads in 200 μl of DRIP elution Buffer (50 mM Tris-HCl, pH 8.0, 10 mM EDTA, 1% sodium dodecyl sulfate (SDS)) by shaking at 65 °C for 30 min at 1400 r.p.m. (Benchmark Scientific, MultiTherm shaker H5000-H). The beads were removed from the supernatant by another round of separation with a magnetic stand and centrifugation. The supernatant was treated with 80 μg of proteinase K (Roche) in the presence of 160 U of RNasin Plus inhibitor for 30 min at 42 °C. RNA:DNA hybrids were purified using QIAquick Nucleotide Removal Kit (Qiagen) and eluted in 200 μl of buffer containing 5 mM Tris-HCl pH 8.5. For RNase H treatment, 50 μg of sonicated genomic DNA was preincubated with 25 U of *E.coli*-RNase H (NEB) overnight at 37 °C. Recovery of RNA:DNA hybrids and RNase H treatment were evaluated by quantitative PCR analysis of spike RNA:DNA duplex. Oligonucleotides used as spike RNA:DNA duplexes are listed in Supplementary Data 1.

### qPCR analysis of DRIP products

Two microliters of DRIP products was used for qPCR with Power SYBR Green PCR Master Mix (Thermo Fisher Scientific) and QuantStudio 6 Flex Real-Time PCR System (Applied Biosystems, QuantStudio Real-Time PCR software). Primers used are listed in Supplementary Data 1.

### Dot blot analysis of DRIP products

DRIP products or whole genomic DNA (10 μl) were mixed with 15 μl of 0.13 N NaOH/3.3 mM EDTA solution and incubated at 90 °C for 10 min. The denatured DRIP products were diluted in 100 μl of 6× SSC and spotted onto a Hybond N+ using Bio-Dot Apparatus. The membrane was cross-linked with UV (0.24 J) and pre-hybridized with ULTRAhyb Ultrasensitive Hybridization Buffer (Invitrogen) overnight at 42 °C. 5′-^32^P-labeled DNA or LNA-oligonucleotide probe was added to the hybridization buffer and incubated overnight at 42 °C. The membrane was washed three times with 2× SSC/0.1% SDS solution for 15 min at 42 °C or 50 °C. A fraction of DRIP samples (5%) was spotted as an input control onto the membrane. Hybridized probe signals were detected using Typhoon RGB Imager (GE Healthcare, Amersham Typhoon Control software). Oligonucleotides used as probe and washing temperature are also listed in Supplementary Data 1. Consensus α-satellite, *Alu*, and *LINE1* probes were hybridized and washed at 42 °C. Using these less stringent hybridization and washing conditions, these probes target variations known to exist within sub-family members of each repetitive element. In particular, the *Alu* consensus probe is 44 nucleotides, so it can recognize all *Alu* subfamilies, except *Alu* that is missing the 3′ region. Therefore, consensus *Alu* and *LINE1* probes recognize ~11% and 18% of the human genome, respectively.

### RNA strand analysis of DRIP products

Fifty micrograms of genomic DNA prepared as described above was diluted in 250 μl of sonication buffer and sonicated using Sonicator W-220 (20 cycles at Lv3.5, 10 s ON/40 s OFF). During sonication, samples were kept cold very carefully. A fraction of the genomic DNA sample (90%, 225 μl) was used for immunoprecipitation with the S9.6 antibody (Sigma or Kerafast), and the remaining fraction (10%, 25 μl) was used as the input control. Protein A beads (Dynabeads Protein A, Invitrogen) (100 μl) were blocked with 0.5% BSA in PBS containing 5 mM EDTA overnight at 4 °C. After washing twice with DRIP buffer, 20 μg of S9.6 antibody or control mouse IgG (sc-2025, Santa Cruz) was applied to the blocked Dynabeads in DRIP buffer overnight at 4 °C. After washing twice with DRIP buffer, the beads were resuspended in 100 μl of DRIP buffer containing 500 U of RNasin Plus inhibitor (Promega). Sonicated DNA was diluted to half concentration in a buffer containing 100 mM Tris-HCl pH 7.4, 10 mM EDTA, 2% NP-40, and 0.2% sodium deoxycholate. Then, 100 μl of the beads was added to the DNA solution and incubated overnight at 4 °C with rotation. The beads were washed by the following steps: (1) twice with DRIP buffer; (2) twice with DRIP High buffer; (3) twice with DRIP Li buffer; (4) one with DRIP TE NaCl+ buffer; and (5) twice with 100 mM Tris-HCl pH 8.0 buffer. After removal of 100 mM Tris-HCl pH 8.0 buffer using a magnetic stand, the beads were treated with 10 U of TURBO DNase (Thermo Fisher Scientific) at 37 °C for 1 h in 100 μl of TURBO DNase buffer containing 160 U of RNasin Plus inhibitor. After adding 100 μl of double concentration DRIP elution buffer (100 mM Tris-HCl, pH 8.0, 20 mM EDTA, 2% SDS), RNAs were eluted from the beads by shaking at 65 °C for 30 min at 1400 r.p.m. (Benchmark Scientific, MultiTherm shaker H5000-H). The beads were removed from the supernatant by another round of separation with a magnetic stand and centrifugation. The supernatant was treated with 80 μg of proteinase K (Roche) for 30 min at 42 °C. RNAs were purified using QIAquick Nucleotide Removal Kit (Qiagen) and eluted in 100 μl of buffer containing 5 mM Tris-HCl pH 7.6. For RNase H treatment, 50 μg of sonicated genomic DNA was preincubated with 25 U of *E. coli*-RNase H (NEB) overnight at 37 °C. The input control was treated with RNase I and TURBO DNase and was purified using QIAquick Nucleotide Removal Kit.

DRIP products were incubated at 80 °C for 10 min. The denatured DRIP products were diluted in 100 μl of 6× SSC and spotted onto a Hybond N+ using Bio-Dot Apparatus. The membrane was cross-linked with UV (0.24 J) and pre-hybridized with ULTRAhyb Ultrasensitive Hybridization Buffer (Invitrogen) overnight at 42 °C. 5′-^32^P-labeled LNA-oligonucleotide probe was added to hybridization buffer and incubated overnight at 42 °C. The membrane was washed three times with 2× SSC/0.1% SDS solution for 15 min at 42 °C or 55 °C. Hybridized probe signals were detected using a Typhoon RGB Imager (GE Healthcare, Amersham Typhoon Control software). A fraction of DRIP samples (5%) was spotted onto the membrane. Oligonucleotides used as probes and washing temperatures are listed in Supplementary Data 1.

### Preparation of duplex substrates

Sense or antisense oligonucleotides of telomere sequences were purchased from IDT and Dharmacon. The 5′ ends of RNA and DNA strands to be analyzed were biotinylated. Sense and antisense oligonucleotides were annealed in annealing buffer (10 mM Tris-HCl pH 7.5, 50 mM NaCl) to prepare perfectly matched or mismatched dsRNAs or RNA:DNA hybrids, which were used as substrates for in vitro editing assay.

### Preparation of recombinant ADAR1 proteins

All procedures were carried out at 4 °C. HAT-ADAR1p110-WT-, FLAG-ADAR1p110-WT-, or HA-ADAR1p110-EAA-expressing Sf9 cells were prepared with baculovirus^69^. The cells were washed with PBS and resuspended in Tris+ buffer (250 mM Tris pH 7.8, 1 mM dithiothreitol (DTT), 0.6 mM phenylmethylsulfonyl fluoride (PMSF), proteinase inhibitor cocktail). The cells were sonicated and debris was removed by centrifugation. The supernatant (cell extract) was diluted with an equal volume of 2× TGK buffer (100 mM Tris-HCl pH 7.8, 200 mM NaCl, 40% glycerol, 1 mM DTT, 0.6 mM PMSF, proteinase inhibitor cocktail) and stored at −80 °C.

HAT-ADAR1p110-WT was purified using TALON Metal Affinity Resin (Clontech). The resin was prewashed with STD300 buffer (50 mM Tris-HCl pH 7.0, 300 mM NaCl, 20% glycerol, 1 mM β-mercaptoethanol, 0.05% NP-40). After buffer exchange to STD300 using Zeba^TM^ 7 K molecular weight cut-off (MWCO) spin desalting column (Thermo Fisher), the cell extract was loaded onto the resin. After washing with STD300 buffer, the resin was treated with 80 kU of micrococcal nuclease (NEB) for 30 min in STD300 buffer containing 1 mM CaCl_2_ at room temperature and washed with STD300 buffer containing 0.5 mM EDTA and 0.5 mM EGTA and then washed with STD300 buffer containing 7.5 mM imidazole. HAT-ADAR1p110-WT recombinant protein was eluted with STD300 buffer containing 150 mM imidazole and proteinase inhibitor cocktail. Imidazole was removed by using Zeba^TM^ 7 K MWCO spin desalting column.

FLAG-ADAR1p110-WT or HA-ADAR1p110-EAA was purified using anti-FLAG M2 agarose (Sigma) or anti-HA agarose (Thermo Fisher Scientific), respectively. The agarose was washed with STD150 buffer (50 mM Tris-HCl pH 7.0, 150 mM NaCl, 20% glycerol, 1 mM β-mercaptoethanol, 0.05% NP-40). After buffer exchange to STD150 using Zeba^TM^ 7 K MWCO spin desalting column, the cell extract was loaded onto the agarose. After washing with STD150 buffer, the agarose was treated with 80 kU of micrococcal nuclease (NEB) for 30 min in STD150 buffer containing 2 mM CaCl_2_ at room temperature and washed with STD150 buffer containing 3 mM EDTA and 3 mM EGTA, STD150 buffer, STD500 buffer (50 mM Tris-HCl pH 7.0, 500 mM NaCl, 20% glycerol, 1 mM β-mercaptoethanol, 0.05% NP-40), and again STD150 buffer. FLAG-ADAR1p110-WT or HA-ADAR1p110-EAA recombinant protein was eluted with STD150 buffer containing protease inhibitor cocktail and 0.1 mg/ml FLAG peptide or HA peptide, respectively.

All recombinant proteins purified were stored in STD150 buffer containing 1 mM DTT, instead of 1 mM β-mercaptoethanol, at −80 °C

### In vitro editing assay

The in vitro editing reaction mixture, containing 5 nM of telomere RNA:RNA duplex substrates and 75 nM of HAT-ADAR1p110-WT, FLAG-ADAR1p110-WT, or HA-ADAR1p110-EAA protein, was incubated at 37 °C for 2 h in in vitro editing buffer I (20 mM HEPES-KOH pH 7.5, 100 mM NaCl, 0.01% NP-40, 5% glycerol, 1 mM DTT). For editing of RNA:DNA hybrid substrates, in vitro editing buffer II (20 mM HEPES-KOH pH 7.5, 20 mM NaCl, 0.01% NP-40, 5% glycerol, 1 mM DTT) was used. Edited RNA or DNA strands were purified using Dynabeads MyOne Streptavidin C1 (Thermo Fisher Scientific). To remove opposite RNA or DNA strands, RNase H (NEB) or TURBO DNase (Thermo Fisher Scientific) was used, respectively. For sequencing of edited substrates, reverse transcription-PCR was carried out for RNA strands, while PCR was carried out for DNA strands. Each reaction used specific primer sets (Supplementary Data 1). RT reactions were carried out using SuperScript III Reverse Transcriptase (Thermo Fisher Scientific), and PCR reactions were performed using Platinum *Taq* DNA polymerase (Thermo Fisher Scientific). PCR products were sequenced using a specific sequencing primer, and the ratio of A and G peaks in the chromatograms were analyzed by CodonCode Aligner (CodonCode Corporation).

### Protein co-IP

Cells expressing FLAG-ADAR1p110-WT, FLAG-ADAR1p110-EAA, FLAG-ADAR2-WT, FLAG-ADAR2-EAA, or FLAG-RNaseH2A were fixed with 0.3% formaldehyde in PBS containing 1 mM DTT at room temperature for 10 min. After washing twice with PBS, the cells were suspended in co-IP buffer (50 mM Tris-HCl pH 7.6, 150 mM NaCl, 1 mM EDTA, 1% NP-40, 0.5% sodium deoxycholate, 0.1% SDS, proteinase inhibitor cocktail, PhosStop, RNasin Plus inhibitor) and sonicated. The debris was removed by centrifugation and the supernatant was incubated overnight at 4 °C with 50 μl of anti-FLAG M2 magnetic beads (Sigma), prewashed and blocked with 20% BSA blocker in co-IP buffer. The beads were washed three times with co-IP buffer and NP-40 wash buffer (50 mM Tris-HCl pH 7.6, 150 mM NaCl, 1 mM EDTA, 1% NP-40). Laemmli buffer containing proteinase inhibitor cocktail and PhosStop phosphatase inhibitor cocktail was added to the beads and boiled at 98 °C for 10 min. Interacting proteins with ADAR1p110-WT, ADAR2, or RNaseH2A were detected by immunoblot analysis as described above; 2.5% extracts of co-IP products were used as input controls.

### Preparation of RNase H2 complex and RNA:DNA hybrid cleavage assay

To prepare recombinant human RNase H2A/2B/2C triple complex, pET28-FLAG-RNaseH2A and pET15-His-RNaseH2B/2C vectors were co-transformed into *E. coli* BL21 cells cultured in LB medium containing 100 μg/ml ampicillin and 20 μg/ml kanamycin. Protein induction was started at an optical density of 0.6 with 0.4 mM isopropyl β-d-1-thiogalactopyranoside and incubated overnight at 30 °C. After harvesting cells by centrifugation, cells were suspended in 40 mM HEPES pH 7.0, 75 mM NaCl, 5% glycerol, proteinase inhibitor cocktail, and 1 mg/ml lysozyme and then sonicated. The debris was removed by centrifugation twice at 12,000 × *g*. The supernatant was diluted with an equal volume of dilution buffer (40 mM NaH_2_PO_4_ pH 7.0, 500 mM NaCl, 5% glycerol) and mixed with Ni-NTA agarose (Qiagen) at 4 °C. The agarose was washed with STD300 buffer containing 5 mM imidazole. Recombinant RNase H2A/2B/2C complex was eluted with 20 ml of STD300 buffer containing 150 mM imidazole and proteinase inhibitor cocktail. Anti-FLAG M2 affinity gel (Sigma) was washed with STD150 buffer and mixed with an eluted fraction of Ni-NTA agarose at 4 °C. After washing with STD150 buffer, the resin was treated with 80 kU of micrococcal nuclease (NEB) for 30 min in STD150 buffer containing 2 mM CaCl_2_ at room temperature and washed with STD500 buffer and then with STD150 buffer. FLAG-RNase H2A/His-RNase H2B/2C complexes were eluted with 0.1 mg/ml FLAG peptide in STD150 buffer containing proteinase inhibitor cocktail.

5′-^32^P-labeled oligonucleotide RNAs were annealed with complementary DNAs as described above (Supplementary Data 1). Cleavage assays of RNA:DNA hybrid by RNase H2A/2B/2C complex were done in a 50 μl reaction mixture containing 50 mM Tris-HCl pH 8.5, 75 mM KCl, 3 mM MgCl_2_, 10 mM DTT, 2 nM RNase H2A/2B/2C complex, 1 nM RNA:DNA substrate, and RNasin plus inhibitor. Cleavage assays by recombinant human RNase H1 (ab153634, Abcam) were done in a 50 μl reaction mixture containing 25 mM Tris-HCl pH 7.5, 50 mM KCl, 5 mM MgCl_2_, 1 mM DTT, 10 μg/ml BSA, 5 nM RNase H1, 1 nM RNA:DNA substrate, and RNasin plus inhibitor. Reaction mixtures were incubated at 37 °C and 7.5 μl aliquots were taken after 0, 5, 10, 30, and 60 min. At each time point, to stop the reaction, gel loading buffer (80% formamide, 20% glycerol, 0.025% bromophenol) was added to the aliquots. After heating at 80 °C for 10 min, samples were analyzed by 10% Urea-PAGE. 5′-^32^P-labeled RNA signals were detected using a Typhoon RGB Imager (GE Healthcare, Amersham Typhoon Control software).

### Telomere FISH analysis

Exponentially growing cells were treated with colcemid (60 ng/ml) for 1 h and harvested. Then, cells were subsequently swollen in a hypotonic 0.075 M KCl solution for 20 min at room temperature and then fixed in a freshly prepared 3:1 mix of methanol:acetic acid four times. After fixation, cells were dropped onto glass microscope slides and allowed to dry for 2 days at room temperature. The slides were immersed in PBS at 37 °C for 30 min, fixed in 4% formaldehyde in PBS for 2 min, and washed three times with PBS for 5 min. The slides were then treated with 1 mg/ml pepsin solution (pH 2.0) at 37 °C for 2–5 min. After washing with PBS for 10 s, the slides were fixed in 4% formaldehyde in PBS and washed three times with PBS for 5 min. Then, 10 μl of hybridization mixture containing 70% formamide, 1% (w/v) blocking reagent (Roche) in a maleic acid buffer (pH 7.0), and 3 ng of fluorescence-labeled telomeric peptide nucleic acid (PNA) probe FITC-(CCCTAA)_4_ were applied to each slide and mounted under a coverslip. The slides were heated on an aluminum heat block at 80 °C for 3 min and hybridized with PNA probe for 5 h in 37 °C. After hybridization, the slides were washed twice in 70% formamide/10 mM Tris (pH 7.2) for 15 min, followed by washing three times with 50 mM Tris/150 mM NaCl (pH 7.5)/0.05% Tween-20. Finally, DNA was counterstained with Vectashield with DAPI (Vector Lab). The chromosome samples were observed using a fluorescence microscope and digital images were recorded using a CCD camera and LAS X software (Leica).

### Immuno-FISH assay for MEF cells

Cells were seeded onto coverslips and cultured overnight. The adhered cells were washed twice with cold PBS for 5 min and fixed with 4% paraformaldehyde in PBS for 10 min at room temperature. Cells were then washed three times with PBS for 5 min each and permeabilized with ice-cold 0.5% NP-40 in PBS for 10 min on ice. Cells were washed three times with PBS for 5 min each and incubated with anti-phosphorylated histone γH2AX antibody (Millipore), followed by Alexa 488 secondary antibody (Molecular Probes) with 30% Blocker BSA (bovine serum albumin) buffer (Thermo Fisher Scientific). After staining, labeled protein was cross-linked with 4% paraformaldehyde in PBS for 20 min at room temperature. The samples were washed two times with PBS for 5 min and then dehydrated in 70, 90, and 100% ethanol for 3 min each and air-dried. Hybridization mixtures (10 μl) containing 3 ng of fluorescence-labeled telomeric PNA probe were applied to the slide and mounted under a coverslip. The slides were heated for 3 min on a hot plate at 80 °C. After hybridization with a telomeric PNA probe for 5 h, the cells were washed three times with 70% formamide/10 mM Tris (pH 6.8) for 15 min, followed by a 5-min wash with 0.05 M Tris/0.15 M NaCl (pH 7.5)/0.05% Tween-20 and a 5-min wash with PBS. Mounting and microscopic analysis was performed as for the telomere FISH analysis.

### Immuno-FISH assay for HeLa cells

Cells were seeded onto collagen-coated Ibidi 3-well chambers and transfected with siControl or siADAR1-1. The adhered cells were washed twice with PBS and fixed with 4% paraformaldehyde (Thermo Fisher Scientific) in PBS at room temperature for 10 min. After washing twice with PBS, the cells were permeabilized with 0.25% Tween-20 in PBS. After washing twice with PBS, the cells were treated with 20 μg of RNase A and 500 U of RNase I in PBS at 37 °C for 1 h. After washing three times with PBS, the cells were blocked with 30% Blocker BSA buffer (Thermo Fisher Scientific) for 30 min and incubated with anti-γH2AX antibody (Abcam), followed by Alexa 488 or 647 secondary antibody (Thermo Fisher Scientific) with 30% Blocker BSA buffer (Thermo Fisher Scientific). After staining, labeled protein was cross-linked with 4% paraformaldehyde in PBS at room temperature for 10 min. After washing twice with PBS, the cells were dehydrated in 70%, 85%, and 100% ethanol for 2 min each and air-dried. Fifty microliters of hybridization mixture containing 500 nM TelC-Cy3 PNA probe (PNA bio), 20 mM Tris-HCl pH 7.4, 60% formamide, and 0.5% blocking reagent (Roche) was applied to the chamber. The chamber was heated for 3 min on a heat block at 80 °C. After hybridization with TelC-Cy3 PNA probe at room temperature for 5 h, the cells were washed three times with PBS at 45 °C for 10 min. Finally, DNA was counterstained with DAPI. The slides were mounted with ProLong Gold (Thermo Fisher Scientific). Microscopic images were obtained by using a Leica TCS SP5 DMI6000 CS Confocal Microscope (Leica). Fluorescent images were captured with a ×63.0 lens by LAS X software (Leica). Ectopic expression of FLAG-ADAR1p110 was evaluated by immunostaining with anti-FLAG M2 antibody.

### Chromosome orientation-FISH

Cells were cultured in a medium containing a 3:1 ratio of 5′-bromo-2′-deoxyuridine (BrdU, Sigma):5′-bromo-2′-deoxycytidine (BrdC, Sigma) at a total final concentration of 10 μM during the final 24 h. Colcemid addition led to the accumulation of mitotic cells. Cultures were trypsinized and then treated with hypotonic KCl, fixed, and dropped onto microscope slides. Prior to hybridization of the single-stranded telomere probe (as above for FISH), slides were treated with 0.5 mg/ml RNase A (Sigma) for 10 min at 37 °C and then stained with 0.5 μg/ml Hoechst 33258 (Sigma) in 2× SSC for 15 min at room temperature. Slides were then exposed to 365 nm UV light for 25 min. The BrdU/BrdC-substituted DNA strands were digested with 3 U/μl of exonuclease III (Promega) in a buffer supplied by the manufacturer (50 mM Tris-HCl, 5 mM MgCl_2_, and 5 mM dithiothreitol, pH 8.0) for 10 min at room temperature. An additional denaturation was performed in 70% formamide, 2× SSC at 70 °C for 1 min, followed by dehydration in a cold ethanol series (70, 85, and 100%). The CO-FISH procedure results in the original parental strands serving as single-stranded chromosomal target DNA for hybridization of single-stranded probes.

### Time-lapse imaging

HeLa cells were treated with CellLight Tubulin-GFP and BacMam 2.0 (Thermo Fisher Scientific) for 12 h before siRNA transfection. Transfection of siRNAs into HeLa cells at 1 nM concentration was carried out as described above. Nuclei were visualized by staining of DNA with SiR-DNA reagent (Cytoskeleton) (0.25 μM) for 6 h. Cells were cultured in CellView 3.5 cm glass-bottomed dishes (Greiner). Time-lapse images were obtained using a Leica TCS SP5 DMI6000 CS Confocal Microscope between 48 and 72 h post transfection.

### Statistics and reproducibility

All experiments were performed at least twice or more independent times with similar results. Image quantitation was done using Image J or ImageQuant software (GE Healthcare). Data were analyzed using Microsoft Excel (Microsoft Corporation) and were presented as means ± SD or SEM. Two-tailed *t* tests were conducted where the minimum level of significance was *P* < 0.05.

### Reporting summary

Further information on research design is available in the Nature Research Reporting Summary linked to this article.

## Supplementary information

Supplementary Information

Supplementary Data 1

Supplementary Data 2

Supplementary Data 3

Supplementary Data 4

Supplementary Movie 1

Supplementary Movie 2

Description of Additional Supplementary Files

Reporting Summary

## Data Availability

Data supporting the findings of this study are available from the corresponding author upon request. Source data are provided with this paper.

## Source data

Source Data
