# Supplementary material for: ADAR1 RNA editing enzyme regulates R-loop formation and genome stability at telomeres in cancer cells
Source: Nat Commun. 2021 Mar 12;12:1654. doi: 10.1038/s41467-021-21921-x (PMC7955049; doi:10.1038/s41467-021-21921-x)
Supplement: Supplementary file 1 — Supplementary Information [file 41467_2021_21921_MOESM1_ESM.pdf]

Supplementary Information

ADAR1 RNA editing enzyme regulates R-loop formation and genome stability at telomeres in cancer cells

Yusuke Shiromoto*, Masayuki Sakurai*, Moeko Minakuchi*, Kentaro Ariyoshi, and Kazuko Nishikura#

The Wistar Institute, 3601 Spruce Street, Philadelphia, Pennsylvania 19104, USA

*These authors contributed equally to this work.

Present Address: Masayuki Sakurai, Research Institute for Biomedical Sciences, Tokyo University of Science, Chiba 278-0022, Japan; Kentaro Ariyoshi, Integrated Center for Science and Humanities, Fukushima Medical University, Fukushima, 960-1295, Japan

#Correspondence: Kazuko Nishikura Tel: +1 215 898 3828; Fax: +1 215 898 3911;
Email: kazuko@wistar.org

Inventory

- Supplementary Figure 1-10
- Supplementary Data 1- 4
- Supplementary Movie 1-2

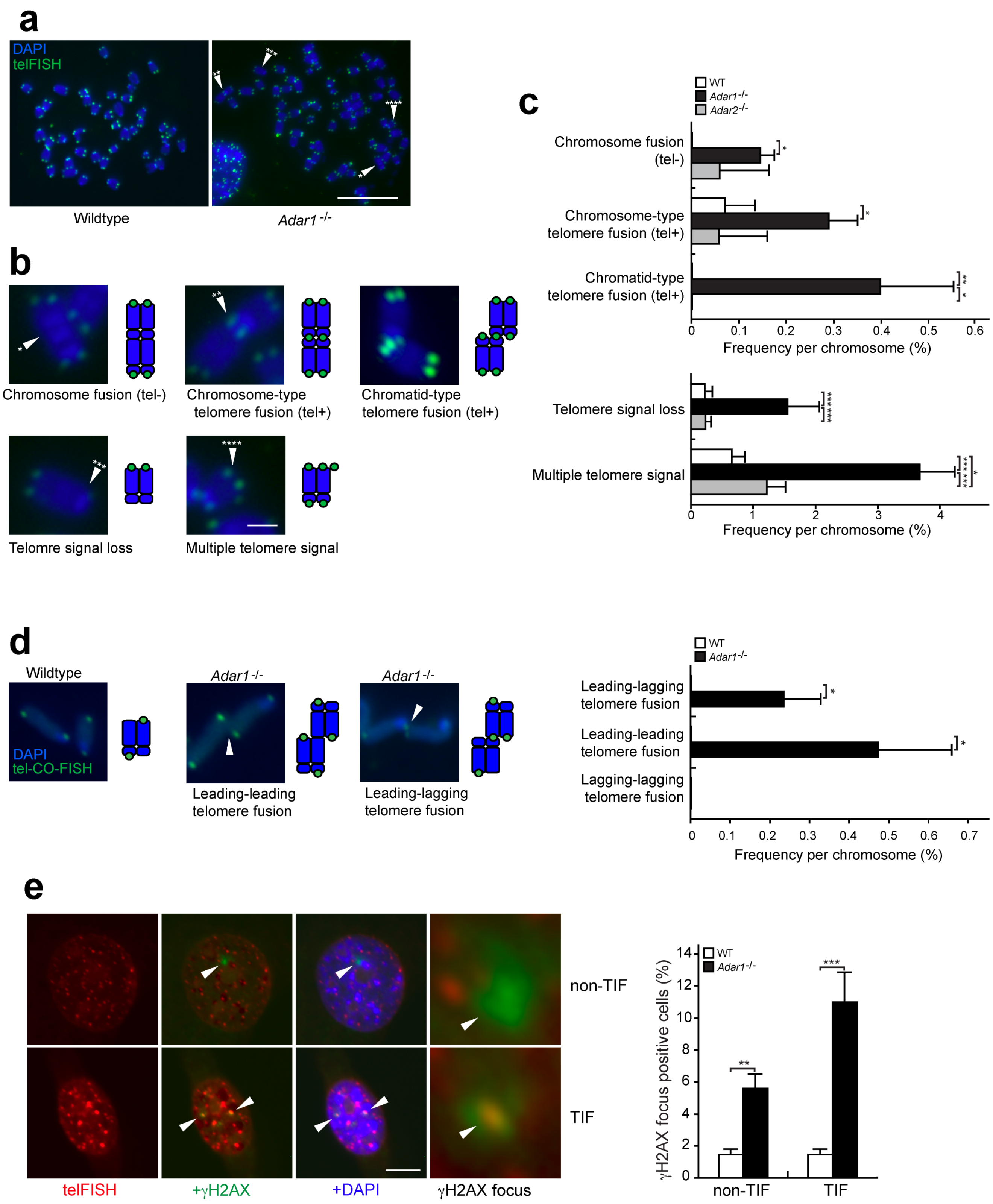
a
DAPI
telFISH
Wildtype
Adar1 -/-
b
Chromosome fusion (tel-)
Chromosome-type
telomere fusion (tel+)
Chromatid-type
telomere fusion (tel+)
Telomre signal loss
Multiple telomere signal
c
WT
Adar1-/-
Adar2-/-
Chromosome fusion
(tel-)
Chromosome-type
telomere fusion (tel+)
Chromatid-type
telomere fusion (tel+)
0 0.1 0.2 0.3 0.4 0.5 0.6
Frequency per chromosome (%)
Telomere signal loss
Multiple telomere signal
0 1 2 3 4
Frequency per chromosome (%)
d
Wildtype
DAPI
tel-CO-FISH
Adar1-/-
Leading-leading
telomere fusion
Leading-lagging
telomere fusion
WT
Adar1-/-
Leading-lagging
telomere fusion
Leading-leading
telomere fusion
Lagging-lagging
telomere fusion
0 0.1 0.2 0.3 0.4 0.5 0.6 0.7
Frequency per chromosome (%)
e
non-TIF
TIF
telFISH
+γH2AX
+DAPI
γH2AX focus
WT
Adar1-/-
γH2AX focus positive cells (%)
0 2 4 6 8 10 12 14
non-TIF
TIF

Supplementary Fig. 1 | Telomere abnormality in *Adar1*$^{-/-}$ MEF cells. **a**, Representative telomere FISH (green) images of metaphase spreads of wild type MEF cells (left) and *Adar1*$^{-/-}$ MEF cells (right) counterstained with DAPI. An arrowhead with '*' indicates chromosome fusion without a telomere signal (tel-), arrowhead with '**' indicates chromosome type telomere fusion (tel+), arrowhead with '***' indicates telomere signal loss, arrowhead with '****' indicates multiple telomere signal. Scale bars, 25 μm. **b**, Magnified image of telomere abnormality in *Adar1*$^{-/-}$ MEF cells. Scale bars, 1 μm. **c**, The frequency of telomere abnormality, chromosome fusion (tel-), chromosome-type telomere fusion (tel+), chromatid-type telomere fusion (tel+), telomere signal loss and multiple telomere signal in wildtype MEF cells, *Adar1*$^{-/-}$ MEF cells and *Adar2*$^{-/-}$ MEF cells. **d**, Telomere fusions detected in *Adar1*$^{-/-}$ MEF cells were further investigated by CO-FISH. Telomere lagging strands were visualized with a FITC-labeled telomeric $(CCCTAA)_4$ PNA probe (green), whereas leading strands remained unmarked. Representative chromosomes with various types of telomere abnormality are shown with schematic drawings. Telomere ends with lagging-lagging and leading-lagging telomere fusions were stained by the FITC probe (green), whereas telomere ends with no staining correspond to leading-leading telomere fusions. White arrow heads indicate the telomere fusion points shown in schematic drawings. **c**, **d**, Quantification of different types of telomere fusions detected: values are given as mean ± standard error with significant differences by two-tailed Student's t-test indicated *, P<0.05 (n = 3, biological replicates). **e**, Immuno-FISH staining of telomeres (red), γH2AX (green) counterstained with DAPI. Upper panels, γH2AX focus (arrowhead) is not co-localized with the telomere signal (non-telomere induced foci: non-TIF) in the nucleus; lower panels, γH2AX focus (arrowhead) is co-localized with telomere signal (telomere dysfunction induced foci: TIF) in the nucleus. The graph indicates the non-TIF positive cells and TIF positive cells in wild type MEF cells and *Adar1*$^{-/-}$ MEF cells. Values are mean ± standard error (n = 3, biologically independent samples) with significant differences by two-tailed Student's t-test indicated **, P<0.01, ***, P < 0.001. Scale bars, 10 μm. **c**-**e**, All individual experimental data values and exact P values are presented in Source Data file.

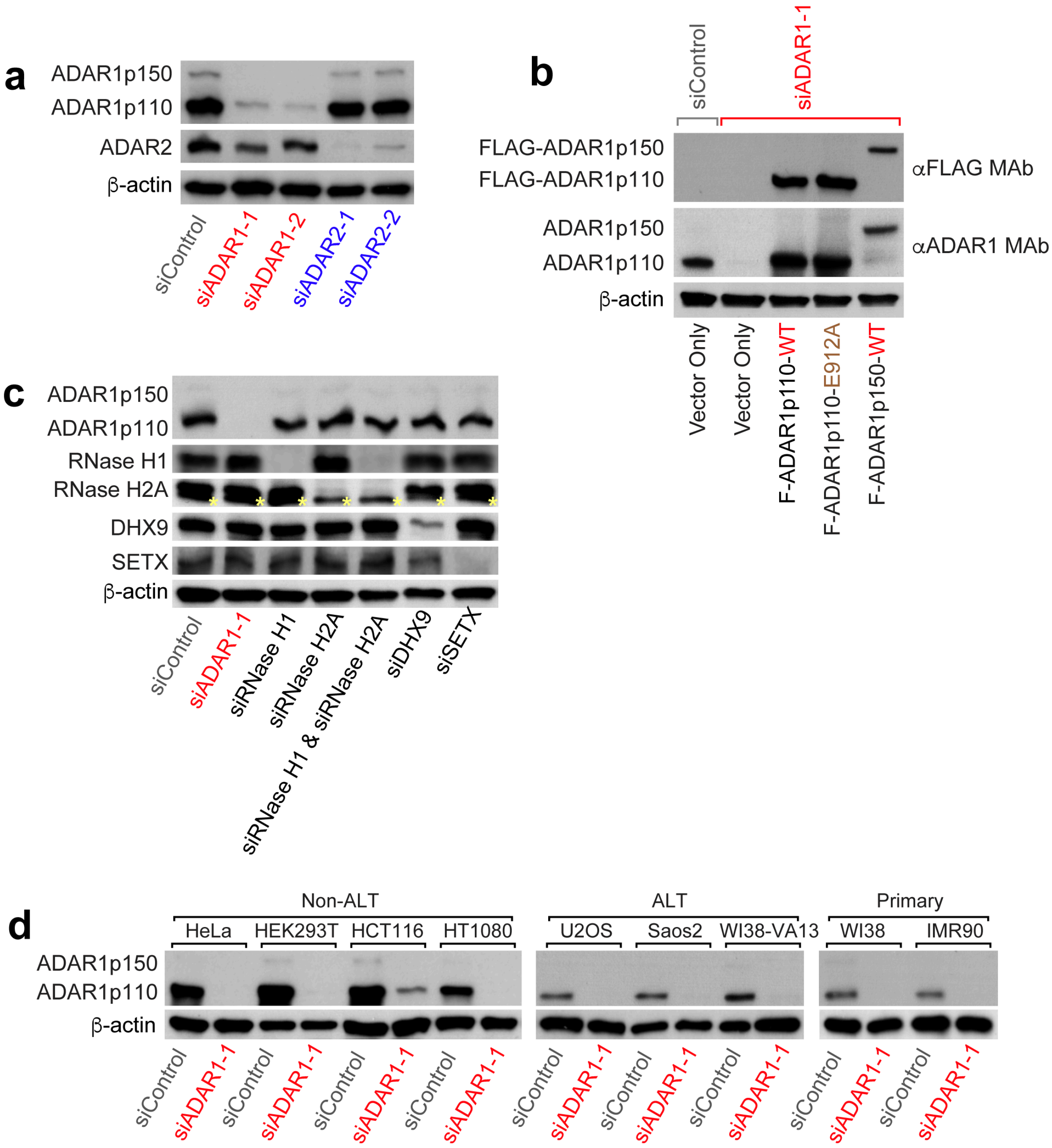

Supplementary Fig. 2 | Validation of siRNA gene knockdown and confirmation of FLAG-ADAR1 expression levels. **a**, ADAR1 and ADAR2 knockdown efficiency by each siRNA was confirmed by immunoblotting. β-actin was used as a loading control. **b**, Lentiviruses for empty vector control or ectopic expression of F-ADAR1p110-WT, F-ADAR1p110-E912A, or F-ADAR1p150-WT were infected in HeLa cells that had been transfected with synthetic siRNAs corresponding to the human ADAR1 mRNA 3'UTR sequence. The expression levels of exogenous FLAG-ADAR1 protein were examined by western blotting analysis using anti-FLAG M2 and anti-ADAR1 antibodies. **c**, R-loop regulator knockdown efficiency by each siRNA was confirmed by immunoblotting. β-actin was used as a loading control. Asterisks show nonspecific bands (RNase H2A panel). **d**, ADAR1 siRNA-mediated knockdown in each cell line was confirmed by immunoblotting. **a-d**, Protein molecular weight markers are presented in Source Data file.

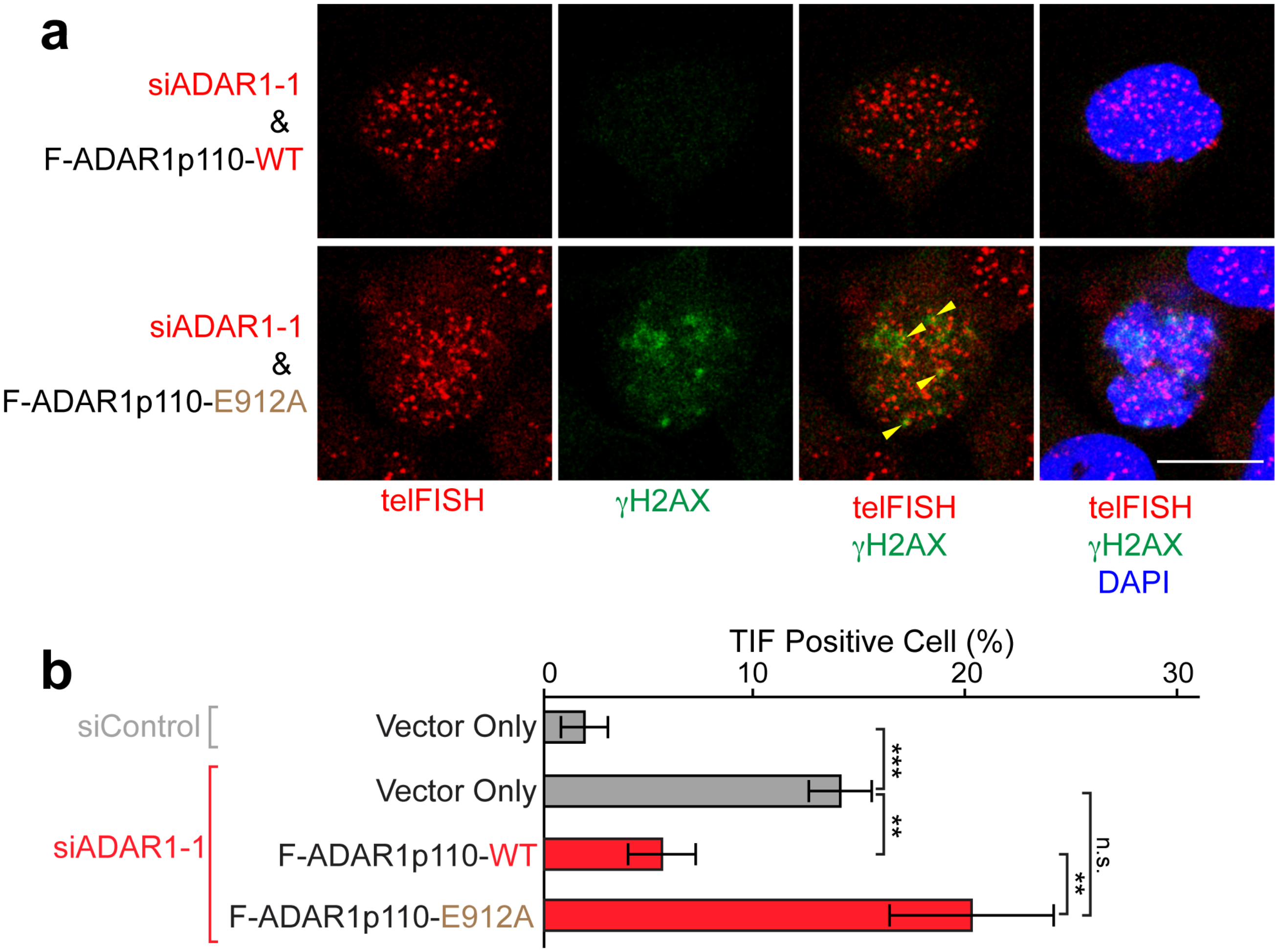

Supplementary Fig. 3 | Ectopic expression of ADAR1p110-WT suppresses TIFs induced in ADAR1 depleted HeLa cells. **a**, Representative Immuno-FISH images of ADAR1 depleted HeLa cells infected with FLAG-ADAR1p110-WT or FLAG-ADAR1p110-E912A mutant lentivirus are shown. Yellow arrowhead indicates TIF. Scale bar, 10 μm. **b**, The frequency of TIFs was estimated by examining at least 200 individual control or ADAR1 depleted HeLa cells infected with empty vector, FLAG-ADAR1p110-WT, or FLAG-ADAR1p110-E912A lentivirus. TIFs induced in ADAR1 depleted HeLa cells were suppressed by FLAG-ADAR1p110-WT but not by ADAR1p110-E912A mutant nor by vector only. HeLa cells with one or more TIFs were counted as TIF positive cells. Values are given as mean ± S.D. (n = 3, biologically independent samples) with significant differences by two-tailed Student's t-test indicated **, P<0.01, ***, P < 0.001, n.s., not significant. All individual experimental data values and exact P values are presented in Source Data file.

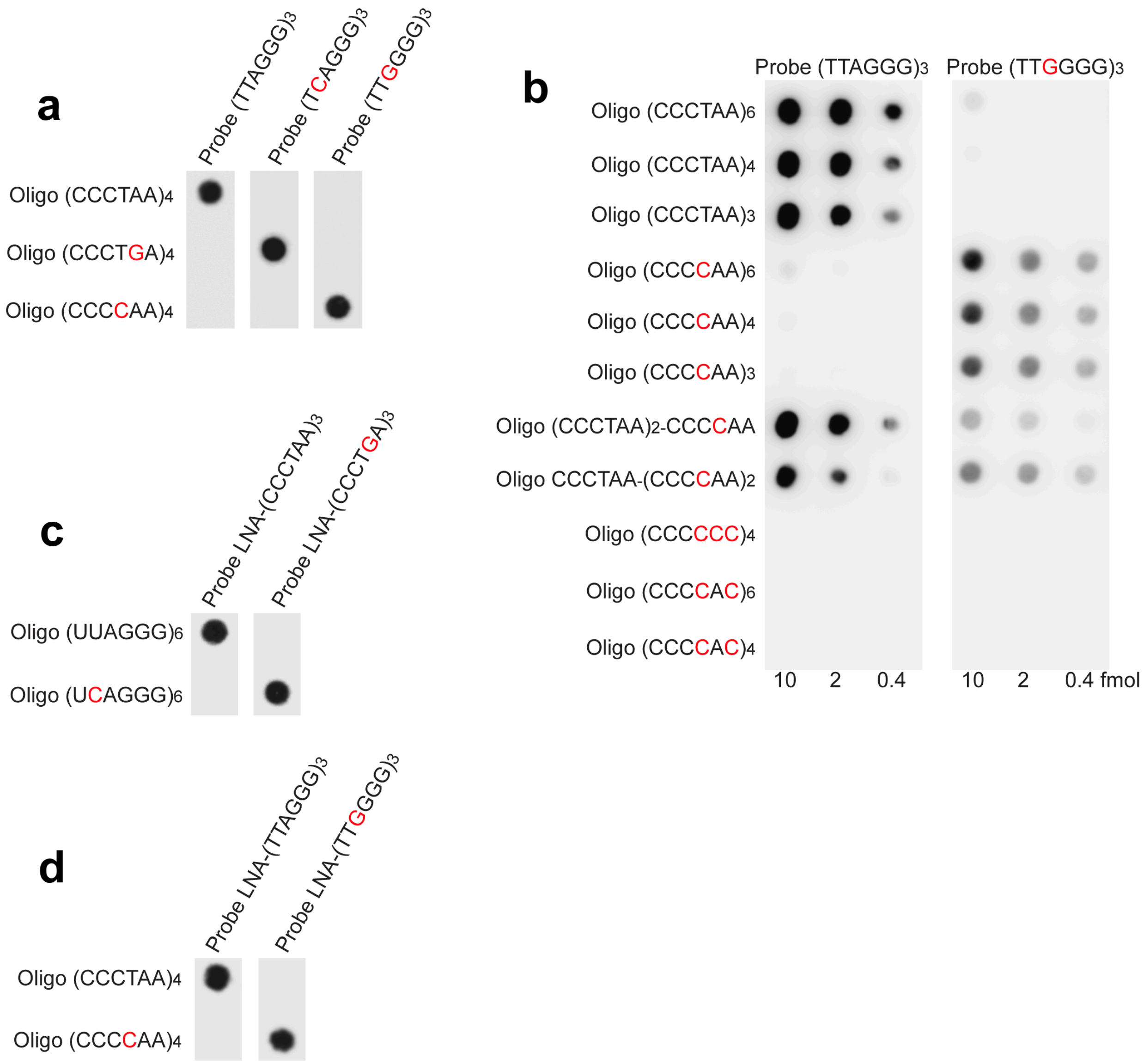

Supplementary Fig. 4 | Validation of telomeric probe specificity. **a**, Specific hybridization of telomeric probes was confirmed using control oligo DNAs. The canonical TTAGGG probes hybridized specifically to CCCTAA oligos but not to CCCTGA or CCCCAA oligos. TCAGGG and TTGGGG variant repeat probes also specifically bound to CCCTGA or CCCCAA oligos, respectively. **b**, Control oligo DNAs were quantitatively detected by the canonical TTAGGG and variant CCCCAA probes. The signals of TTAGGG probes against (CCCTAA)$_2$-CCCCAA oligos were higher than those of CCCTAA-(CCCCAA)$_2$ oligos, whereas the TTGGGG probe signals of (CCCTAA)$_2$-CCCCAA oligos were lower than those of CCCTAA-(CCCCAA)$_2$ oligos. (CCCCCC)$_4$, and (CCCCAC)$_6$ or (CCCCAC)$_4$ oligos were not detected by both TTAGGG canonical and TTGGGG variant probes. Specific hybridization of locked telomeric repeat probes was confirmed using control RNA (**c**) or DNA (**d**) oligos. **c**, The LNA canonical CCCTAA and variant CCCTGA probe hybridized specifically to UUAGGG and UCAGGG RNA oligos, respectively. **d**, The LNA canonical TTAGGG and variant TTGGGG probe hybridized specifically to CCCTAA and CCCCAA DNA oligos, respectively.

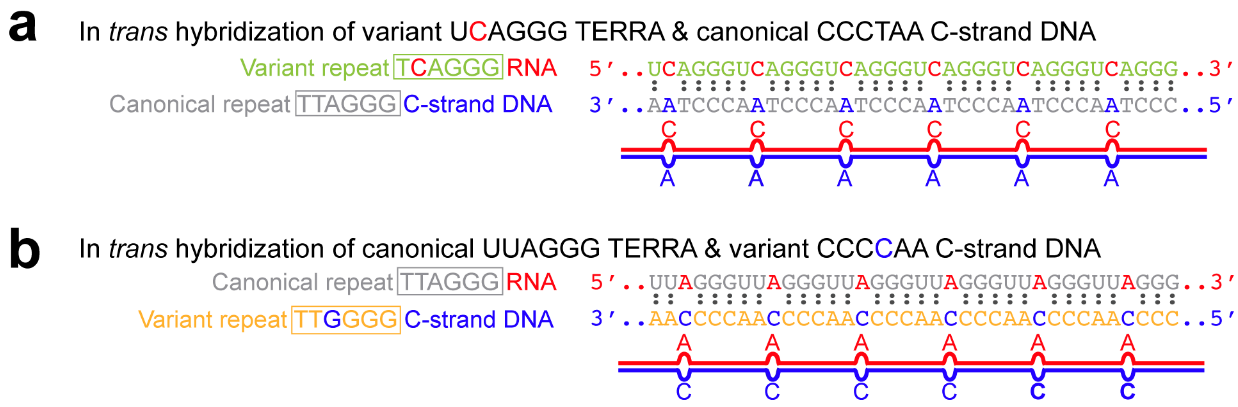

Supplementary Fig. 5 | Formation of telomeric repeat RNA:DNA hybrids containing C-A and A-C mismatches by in *trans* hybridization. **a**, In *trans* hybridization of TERRA RNAs derived from T<u>C</u>AGGG (U<u>C</u>AGGG) variant repeats (green) to the C-strand DNA containing TTAGGG (CCCTAA) canonical repeats (gray), or **b**, TERRA RNAs derived from TTAGGG (UUAGGG) canonical repeats (gray) to the C-strand DNA containing TT<u>G</u>GGG (CCC<u>C</u>AA) variant repeats (orange) results in formation of RNA:DNA hybrids containing C-A and A-C mismatches, respectively.

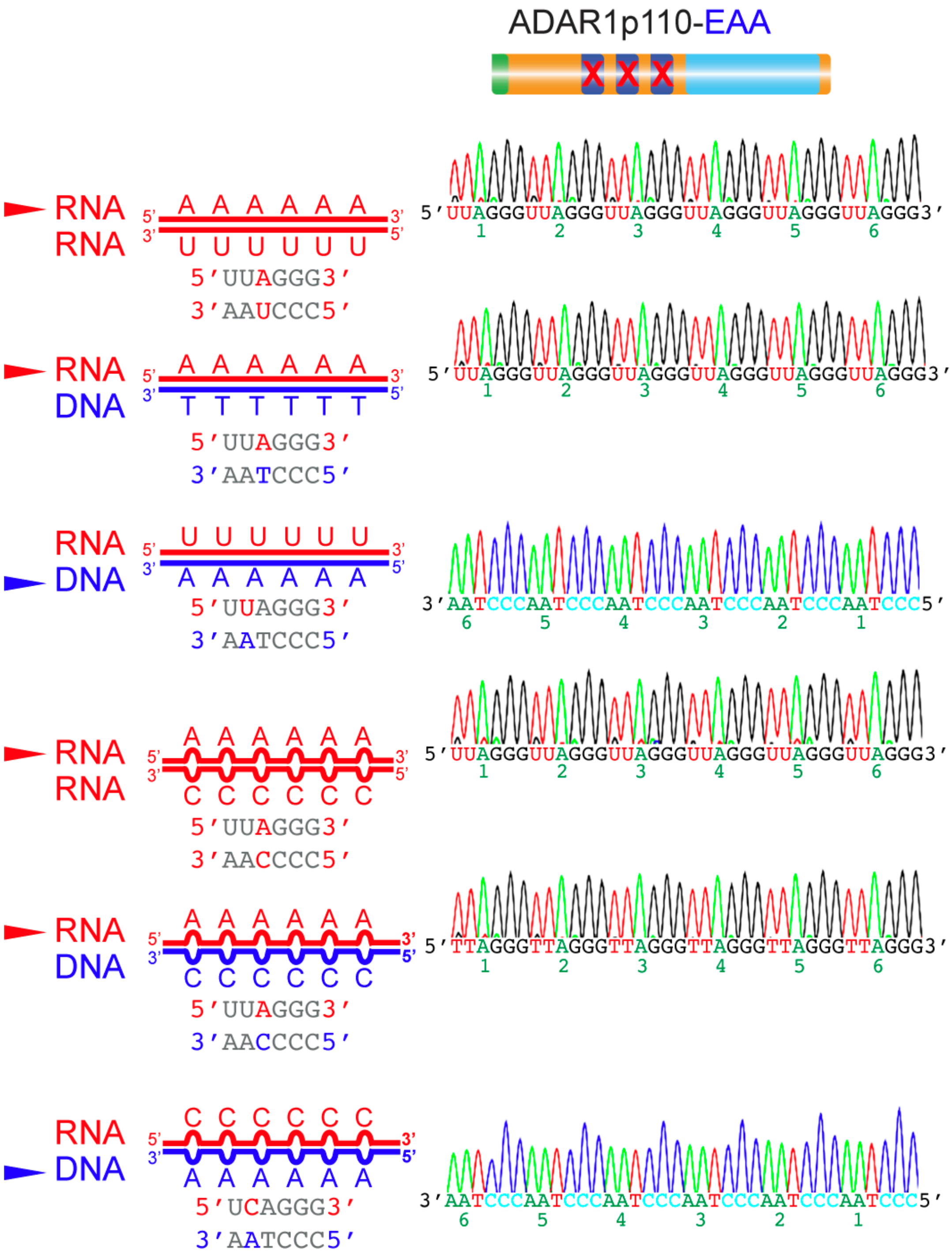

Supplementary Fig. 6 | ADAR1 dsRNA binding domains are required for editing of RNA:DNA hybrids. No editing of RNA:DNA hybrids detected with ADAR1p110-EAA dsRNA binding defective mutant. *In vitro* editing assay was conducted using HA-ADAR1p110-EAA. PCR products (RT-PCR amplified RNA strands and PCR amplified DNA strands) were subjected to Sanger sequencing. The strands analyzed by sequencing are indicated by red and blue arrowheads. ADAR1p110-EAA did not display any editing activity for any of the substrates tested.

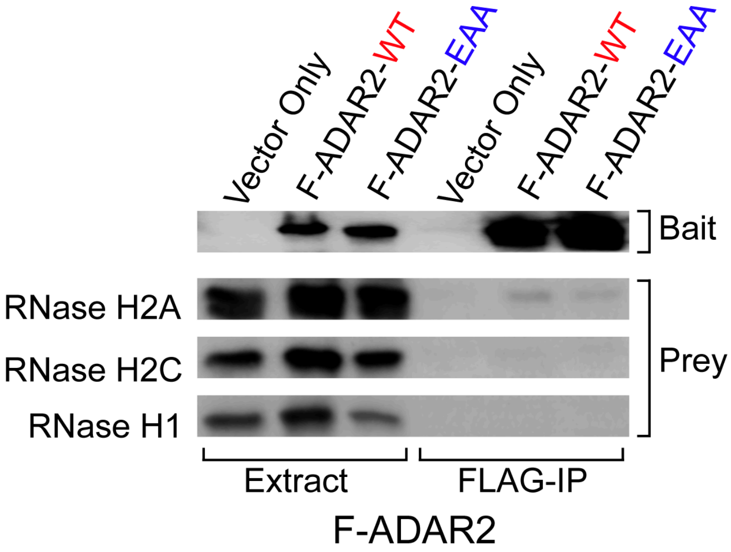

Supplementary Fig. 7 | No interaction between ADAR2 and RNase H1 or RNase H2A and H2C subunits was detected. FLAG-tagged ADAR2 (F-ADAR2-WT and F-ADAR2-EAA) recombinant proteins were purified from permanently transfected HEK293T cell extracts by anti-FLAG M2 magnetic beads. RNase H2 subunits 2A and 2C or RNase H1 were not pulled down with either F-ADAR2-WT or F-ADAR2-EAA. Protein molecular weight markers are presented in Source Data file.

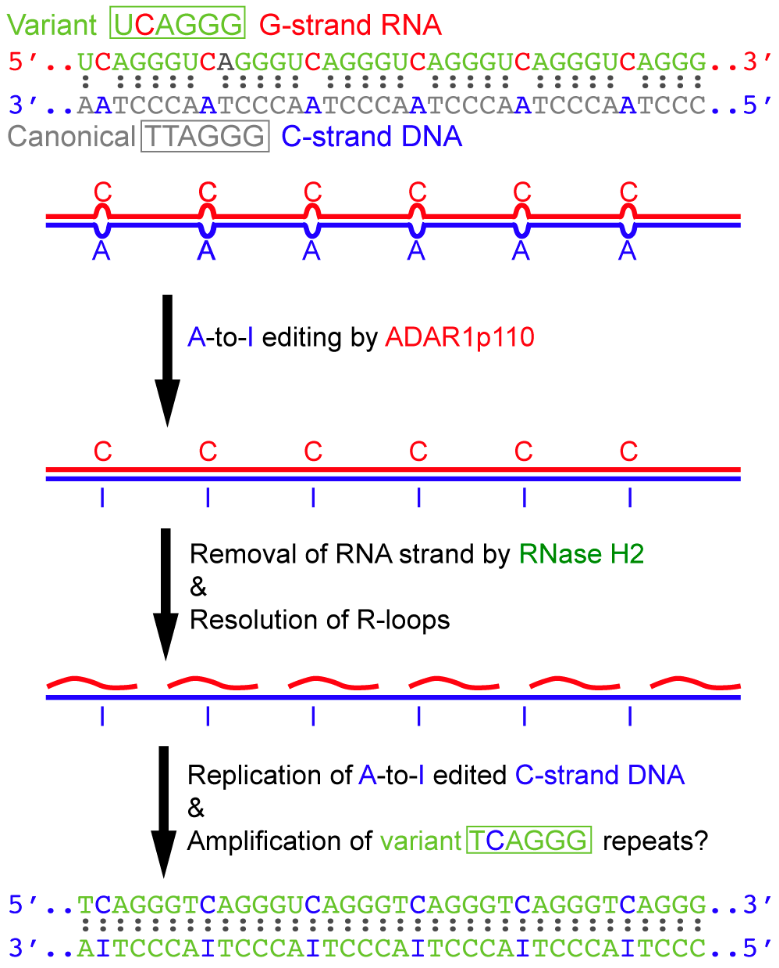

Supplementary Fig. 8 | Expansion model of T<u>C</u>AGGG variant repeat by A-to-I editing.
A-to-I editing of C-A mismatches to C:I base pairs in RNA:DNA hybrids formed between variant and canonical repeats facilitated digestion of RNA strands by RNase H2A/2B/2C triple complexes. Replication of A-to-I edited C-strand DNA may further amplify variant T<u>C</u>AGGG repeats.

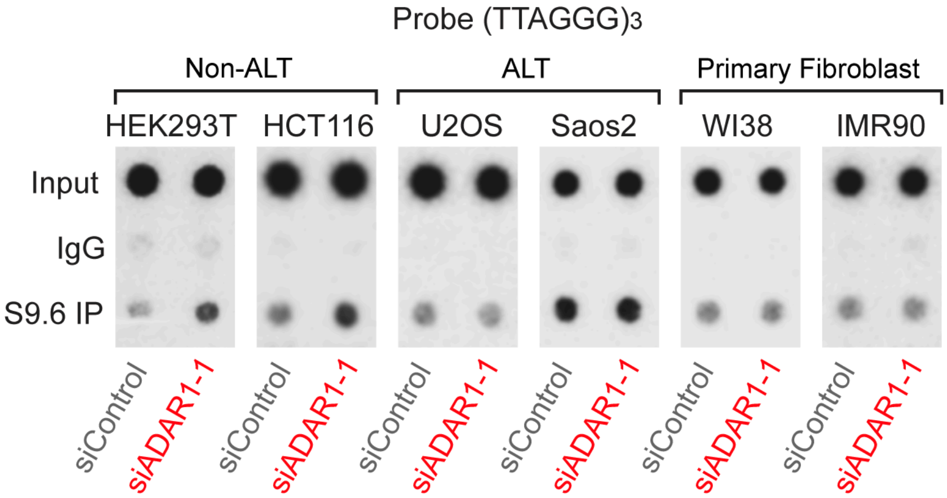

Supplementary Fig. 9 | Detection of RNA:DNA hybrids containing telomeric repeats in ADAR1 depleted non-ALT, ALT, and primary fibroblast cells. DRIP products were examined for C-strand DNAs of telomeric canonical TTAGGG (CCCTAA) repeats by dot blot analysis using an oligonucleotide probe capable of distinguishing a single-nucleotide mismatch (Supplementary Fig. 3a). Two additional similar dot blot analyses were carried out (Source Data file).

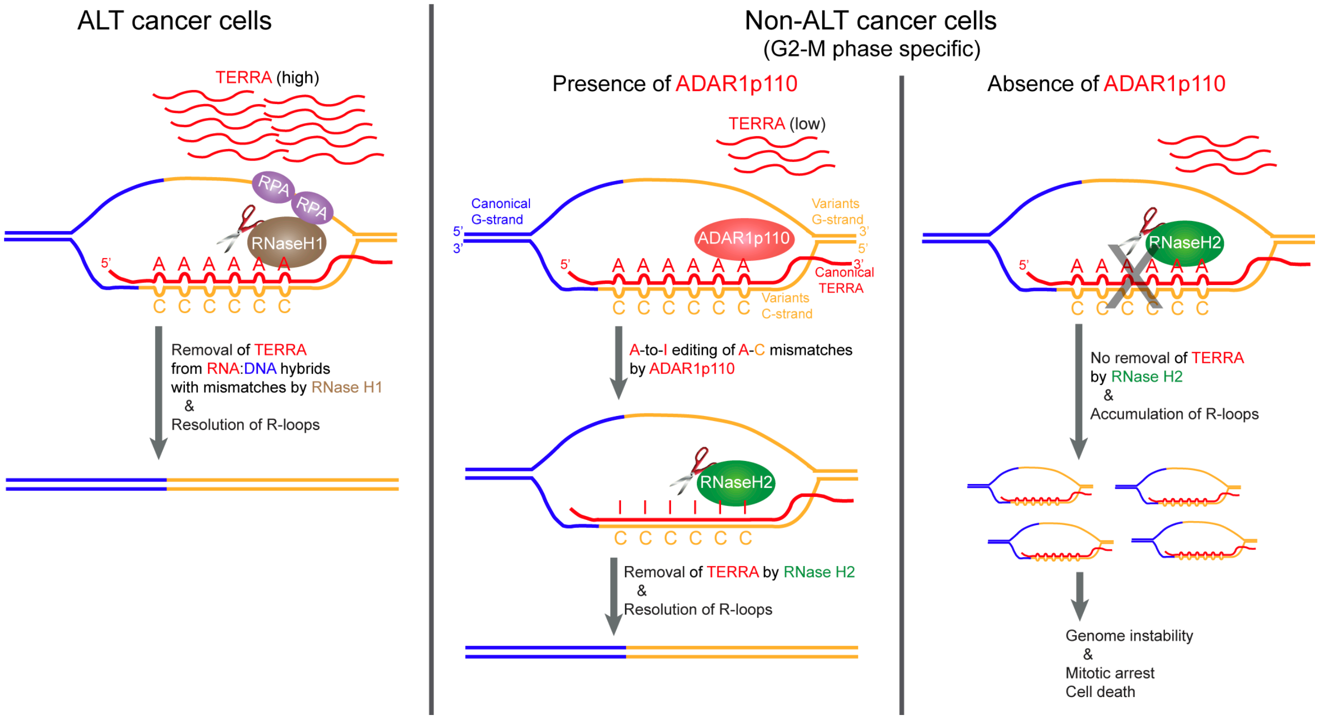

Supplementary Fig. 10 | ADAR1 together with RNase H2 resolves telomeric R-loops in non-ALT cancer cells by editing A-C mismatches of RNA:DNA hybrids formed between canonical and variant repeats. Telomeric variant repeats, wide spread in both ALT and non-ALT cancer cells, cause formation of RNA:DNA hybrids containing A-C mismatches. Unlike RNase H1, RNase H2 cannot degrade the RNA strands of these mismatch-containing RNA:DNA hybrids. Recruitment of RNase H1, perhaps guided by RPA protein bound to single-stranded G-strand DNAs[40], and efficient resolution of telomeric R-loops by RNase H1 have been reported in ALT cells[52]. Close and specific interaction of ADAR1p110 with RNase H2, but not RNase H1, is detected only in non-ALT cells. In non-ALT cells, upregulated ADAR1p110 edits these A-C mismatches to I:C matched base pairs, which is essential for removal of the RNA strands by RNase H2. The cell cycle specific (G2-M) function of RNase H2 has been recently reported[57]. Thus, the R-loop regulatory function of ADAR1p110 may be exerted during G2-M phase. In the absence of ADAR1p110, non-ALT cancer cells die due to genome instability caused by accumulation of telomeric R-loops and mitotic arrest.

Supplementary Data

Supplementary Data 1. DNA and RNA oligos used in this study.

Supplementary Data 2. Data source and statistical analysis for *in vitro* editing assay.

Supplementary Data 3. Data source and statistical analysis for DNA:RNA hybrid cleavage assay.

Supplementary Data 4. Antibodies used in this study.

Supplementary Movies

Supplementary Movie 1. Time-lapse movie of control knockdown HeLa cells. HeLa cells were treated with CellLight Tubulin-GFP and SiR-DNA regent. Time-laps images were obtained between 48-72 hrs after control siRNA transfection. Scan field is 200 x 200 µm.

Supplementary Movie 2. Time-lapse movie of ADAR1 knockdown HeLa cells. HeLa cells were treated with CellLight Tubulin-GFP and SiR-DNA regent. Time-laps images were obtained between 48-72 hrs after ADAR1 siRNA transfection. Scan field is 200 x 200 µm.
