# Supplementary material for: ADAR1 RNA editing enzyme regulates R-loop formation and genome stability at telomeres in cancer cells
Source: Nat Commun. 2021 Mar 12;12:1654. doi: 10.1038/s41467-021-21921-x (PMC7955049; doi:10.1038/s41467-021-21921-x)
Supplement: Supplementary file 8 — Description of Additional Supplementary Files [file 41467_2021_21921_MOESM8_ESM.docx]

Description of additional supplementary information

**Title:** Supplementary Data 1.

**Description:** DNA and RNA oligos used in this study.

**Title:** Supplementary Data 2.

**Description:** Data source and statistical analysis for in vitro editing assay.

**Title:** Supplementary Data 3.

**Description:** Data source and statistical analysis for DNA:RNA hybrid cleavage assay.

**Title:** Supplementary Data 4.

**Description:** Antibodies used in this study.

**Title: Supplementary Movie 1.**

**Description: Time-lapse movie of control knockdown HeLa cells.** HeLa cells were treated with CellLight Tubulin-GFP and SiR-DNA regent. Time-laps images were obtained between 48-72 hrs after control siRNA transfection. Scan field is 200 x 200 μm.

**Title: Supplementary Movie 2.**

**Description: Time-lapse movie of ADAR1 knockdown HeLa cells.** HeLa cells were treated with CellLight Tubulin-GFP and SiR-DNA regent. Time-laps images were obtained between 48-72 hrs after ADAR1 siRNA transfection. Scan field is 200 x 200 μm.
