# Supplementary material for: ADAR1 RNA editing enzyme regulates R-loop formation and genome stability at telomeres in cancer cells
Source: Nat Commun. 2021 Mar 12;12:1654. doi: 10.1038/s41467-021-21921-x (PMC7955049; doi:10.1038/s41467-021-21921-x)
Supplement: Supplementary file 9 — Reporting Summary [file 41467_2021_21921_MOESM9_ESM.pdf]

nature research

Corresponding author(s): Kazuko Nishikura

Last updated by author(s): Feb 15, 2021

Reporting Summary

Nature Research wishes to improve the reproducibility of the work that we publish. This form provides structure for consistency and transparency in reporting. For further information on Nature Research policies, see our Editorial Policies and the Editorial Policy Checklist.

Statistics

For all statistical analyses, confirm that the following items are present in the figure legend, table legend, main text, or Methods section.

n/a	Confirmed	
☐	☒	The exact sample size (*n*) for each experimental group/condition, given as a discrete number and unit of measurement
☐	☒	A statement on whether measurements were taken from distinct samples or whether the same sample was measured repeatedly
☐	☒	The statistical test(s) used AND whether they are one- or two-sided<br>*Only common tests should be described solely by name; describe more complex techniques in the Methods section.*
☒	☐	A description of all covariates tested
☒	☐	A description of any assumptions or corrections, such as tests of normality and adjustment for multiple comparisons
☐	☒	A full description of the statistical parameters including central tendency (e.g. means) or other basic estimates (e.g. regression coefficient) AND variation (e.g. standard deviation) or associated estimates of uncertainty (e.g. confidence intervals)
☐	☒	For null hypothesis testing, the test statistic (e.g. *F*, *t*, *r*) with confidence intervals, effect sizes, degrees of freedom and *P* value noted<br>*Give P values as exact values whenever suitable.*
☒	☐	For Bayesian analysis, information on the choice of priors and Markov chain Monte Carlo settings
☒	☐	For hierarchical and complex designs, identification of the appropriate level for tests and full reporting of outcomes
☒	☐	Estimates of effect sizes (e.g. Cohen's *d*, Pearson's *r*), indicating how they were calculated

Our web collection on statistics for biologists contains articles on many of the points above.

Software and code

Policy information about availability of computer code

Data collection	Microscopic images were obtained by LAS X 3.3.0.16799 (Leica). Radiation signals were detected by Amersham Typhoon Control Software 2.0.0.6 (GE Healthcare). Real-time qPCR data were acquired using QuantStudio Real-Time PCR Software 1.3 (Thermo Fisher Scientific).
Data analysis	The data were analyzed by Microsoft Excel 16.45 (Microsoft Corporation). Image quantitation was done using Image J 1.53a or ImageQuant software 8.1 (GE Healthcare). Sequencing chromatograms were analyzed by CodonCode Aligner 9.0.1 (CodonCode Corporation).

For manuscripts utilizing custom algorithms or software that are central to the research but not yet described in published literature, software must be made available to editors and reviewers. We strongly encourage code deposition in a community repository (e.g. GitHub). See the Nature Research guidelines for submitting code & software for further information.

Data

Policy information about availability of data

All manuscripts must include a data availability statement. This statement should provide the following information, where applicable:

- Accession codes, unique identifiers, or web links for publicly available datasets
- A list of figures that have associated raw data
- A description of any restrictions on data availability

Source data are provided with this article. Other data supporting the findings of this study are available from the corresponding author upon request.

Field-specific reporting

Please select the one below that is the best fit for your research. If you are not sure, read the appropriate sections before making your selection.

☒ Life sciences ☐ Behavioural & social sciences ☐ Ecological, evolutionary & environmental sciences

For a reference copy of the document with all sections, see nature.com/documents/nr-reporting-summary-flat.pdf

Life sciences study design

All studies must disclose on these points even when the disclosure is negative.

Sample size	Sample size, number of replicates and statistical tests were described in figure legends and methods section. The sample size was chosen based on previous experience in the lab or previously published papers using similar analyses. For examples see Sakurai et al. (Nat Struct Mol Biol, 2017) and Chakraborty et al. (Nat Commun, 2018).
Data exclusions	No samples were excluded from analysis.
Replication	All experiments were performed at least twice or more independent times with similar results.
Randomization	Randomly captured microscopic images were used for nuclear morphometric analysis or Telomere FISH analysis. No randomization was required for other experiments. All experiments were at least duplicated to confirm the results. All samples were treated under the same conditions and compared to relevant controls.
Blinding	No blinding was necessary, because all experiments were at least duplicated to confirm the results.

Reporting for specific materials, systems and methods

We require information from authors about some types of materials, experimental systems and methods used in many studies. Here, indicate whether each material, system or method listed is relevant to your study. If you are not sure if a list item applies to your research, read the appropriate section before selecting a response.

Materials & experimental systems

n/a	Involved in the study	
☐	☒	Antibodies
☐	☒	Eukaryotic cell lines
☒	☐	Palaeontology and archaeology
☒	☐	Animals and other organisms
☒	☐	Human research participants
☒	☐	Clinical data
☒	☐	Dual use research of concern

Methods

n/a	Involved in the study	
☒	☐	ChIP-seq
☒	☐	Flow cytometry
☒	☐	MRI-based neuroimaging

Antibodies

Antibodies used

Antibody Name, (clone) Cat#, Vendor or Reference
Anti-ADAR1 15.8.6.1 (Cho et al., 2003) (Supplier: Kazuko Nishikura)
Anti-GAPDH (14C10) 2218 Cell Signaling Technology
Anti-DNA-PKCs Phos-T2609 (10B1) ab18356 abcam
Anti-DNA-PKCs Whole (Y393) ab32566 abcam
Anti-RPA32 Phos-T21 AF6654-SP Novus
Anti-RPA32 Whole (9H8) ab2175 abcam
Anti-gH2AX ab2893 abcam
Anti-gH2AX 05-636-I (JBW301) Millipore
Anti-Cyclin B1 (V152) #4135 Cell Signaling Technology
Anti-Phos-CDC2 (10A11) #4539 Cell Signaling Technology
Anti-Phos-H3(S10) (D2C8) #3377 Cell Signaling Technology
Anti-RNASEH2A A304-149A Bethyl
Anti-RNASEH2C PA5-66770 Thermo Fisher Scientific
Anti-RNASEH1 15606-1-AP Proteintech
Anti-TRF2 NB110-57130 NOVUS
Anti-ADAR2 1.3.1 (Cho et al., 2003) (Supplier: Kazuko Nishikura)
Anti-b-actin (D6A8) 8457 Cell Signaling Technology
Anti-DHX9 (RNA Helicase A) Ab26271 abcam
Anti-SETX A300-104A-M Bethyl
FLAG (M2) F3165 SIGMA

	Anti-DNA-RNA Hybrid antibody (S9.6) ENH002 Kerafast<br>Anti-DNA-RNA Hybrid antibody (S9.6) MABE1095 SIGMA<br>Peroxidase AffiniPure Donkey Anti-Mouse IgG 715-035-150 Jackson Immuno Research<br>AlexaFluor 488 goat anti-rabbit IgG A-11008 Thermo Fisher Scientific<br>AlexaFluor 647 goat anti-mouse IgG A-21236 Thermo Fisher Scientific
Validation	Validation from supplier website. Antibodies were validated to react with human proteins by immunoblot analysis.<br>Anti-GAPDH, Anti-DNA-PKCs Phos-T2609, Anti-DNA-PKCs Whole, Anti-RPA32 Phos-T21, Anti-RPA32 Whole, Anti-gH2AX, Anti-Cyclin B1, Anti-Phos-CDC2, Anti-Phos-H3(S10), Anti-RNASEH2A, Anti-RNASEH2C, Anti-RNASEH1, Anti-TRF2, Anti-b-actin, Anti-DHX9, Anti-SETX.<br><br>Validation from supplier website. Antibodies were validated to react with N-terminal FLAG fusion proteins by immunoblot analysis and immunofluorescence staining.<br>FLAG (M2) antibody<br><br>Validation from supplier website. Antibodies were validated to react with human or mouse gH2AX proteins by immunofluorescence staining.<br>Anti-gH2AX ab2893 abcam (Human gH2AX)<br>Anti-gH2AX 05-636-I Millipore (Mouse gH2AX)<br><br>Validation from supplier website. Antibodies were validated to react with DNA:RNA hybrids by dot blot analysis.<br>Anti-DNA-RNA Hybrid antibody (S9.6) Kerafast<br>Anti-DNA-RNA Hybrid antibody (S9.6) SIGMA<br>Additionally, S9.6 antibodies were validated to react with DNA:RNA hybrids by DRIP-qPCR using spike DNA:RNA duplexes.<br><br>Secondary antibodies were validated by the manufactures.<br>Peroxidase AffiniPure Donkey Anti-Mouse IgG (Immunoblot analysis)<br>AlexaFluor 488 goat anti-rabbit IgG (Immunofluorescence staining)<br>AlexaFluor 647 goat anti-mouse IgG (Immunofluorescence staining)<br><br>ADAR1 and ADAR2 antibodies were validated by a previous publication (Cho et al., 2003).

Eukaryotic cell lines

Policy information about cell lines

Cell line source(s)	HeLa human ovarian carcinoma (ATCC CCL-2)<br>HEK293T human embryonic kidney (ATCC CRL-11268)<br>HCT116 human colon carcinoma (ATCC CCL-247)<br>HT-1080 human fibrosarcoma (ATCC CCL-121)<br>U2OS human osteosarcoma (ATCC HTB-96)<br>WI38-VA13 human virus-transformed fibroblasts (ATCC CCL-75.1)<br>Saos2 human osteosarcoma (ATCC HTB-85)<br>WI38 lung fibroblast (ATCC CCL-75)<br>IMR90 lung fibroblast cells (ATCC CCL-186)<br>Wild type MEF cells and Adar1-/- MEF cells were established from wild type and Adar1-/- mice, respectively (Wang et al., 2004).<br>Adar2-/- MEF cells were established from Adar2-/- mice (Higuchi et al., 2000).
Authentication	The human cell lines were not authenticated. The human cell lines were purchased from ATCC. MEF cells were authenticated by genotyping PCRs and western blotting.
Mycoplasma contamination	The cell lines were free of mycoplasma contamination. We performed regular plasmocin (Invivogen) treatment of the cell lines.
Commonly misidentified lines<br>(See ICLAC register)	None of the cell lines used are listed in the ICLAC list.
