# Supplementary material for: ADAR1 RNA editing enzyme regulates R-loop formation and genome stability at telomeres in cancer cells
Source: Nat Commun. 2021 Mar 12;12:1654. doi: 10.1038/s41467-021-21921-x (PMC7955049; doi:10.1038/s41467-021-21921-x)

Figure 1

Figure 1d

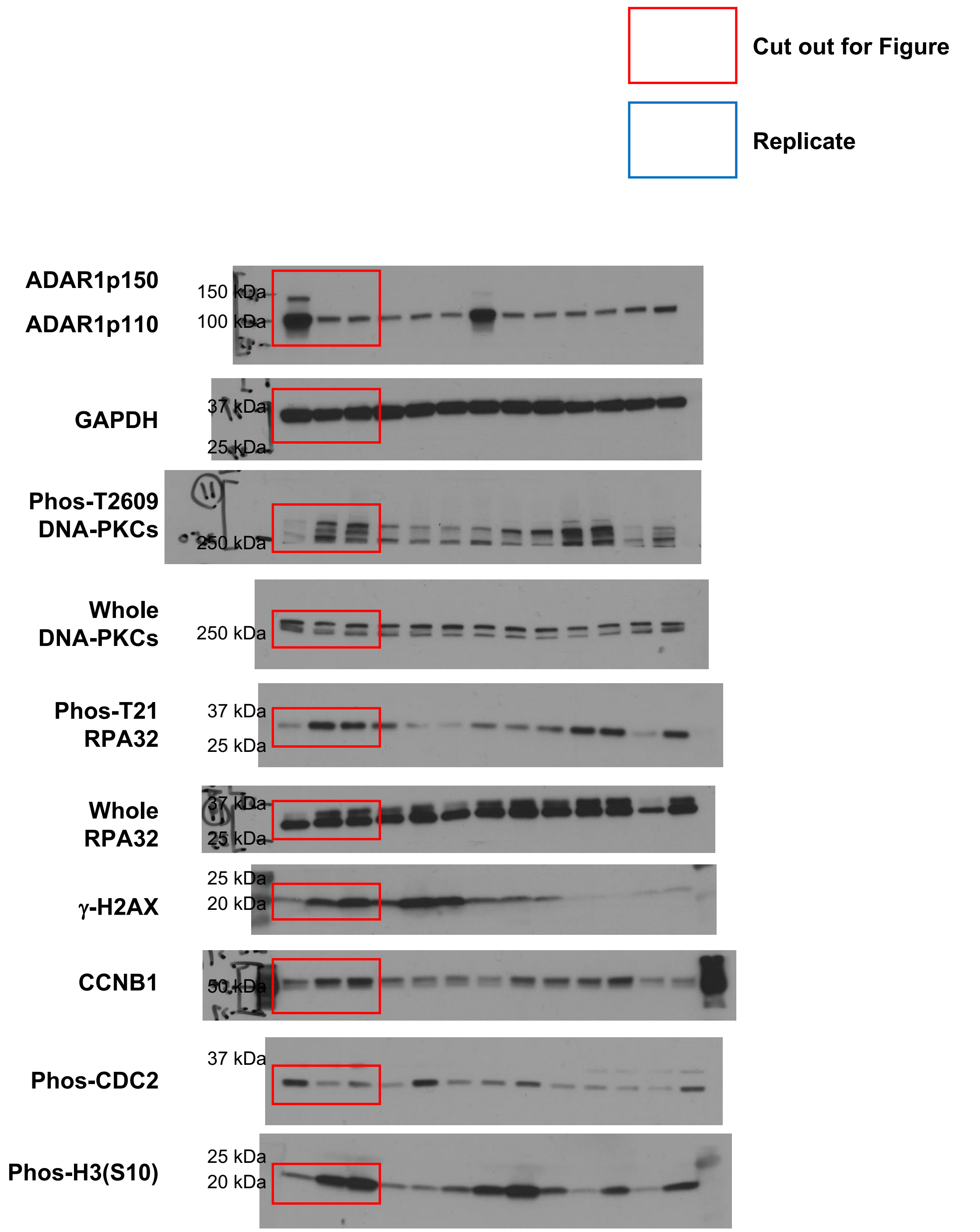

Figure 2

Figure 2a

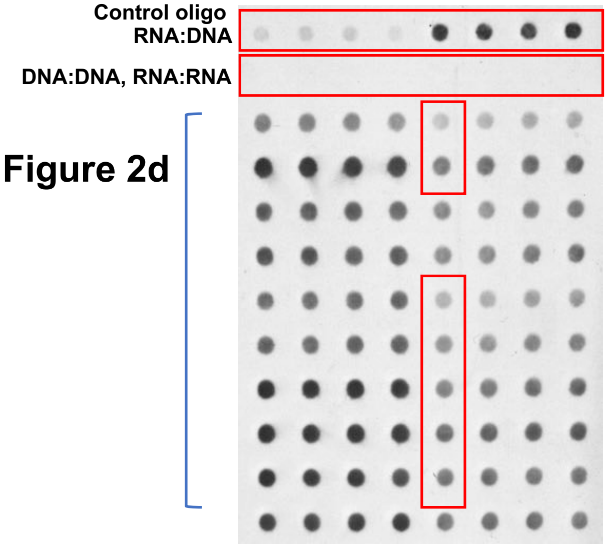

Figure 2b

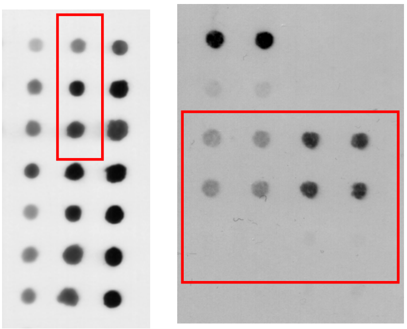

Figure 2c

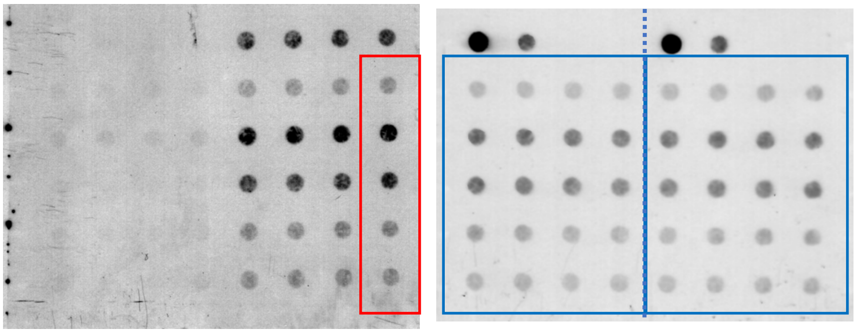

Figure 2d

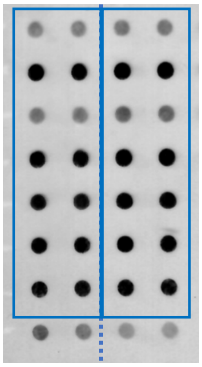

Figure 2e

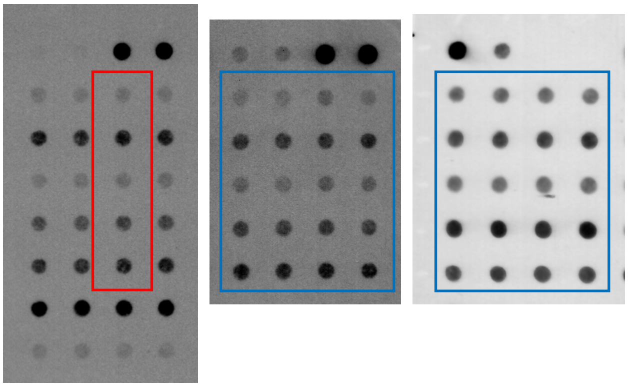

Figure 3

Figure 3b

Probe
Telomere
DNA-(TTAGGG)3

Probe
Alpha-Satellite

Probe
Alu

Probe
LINE1

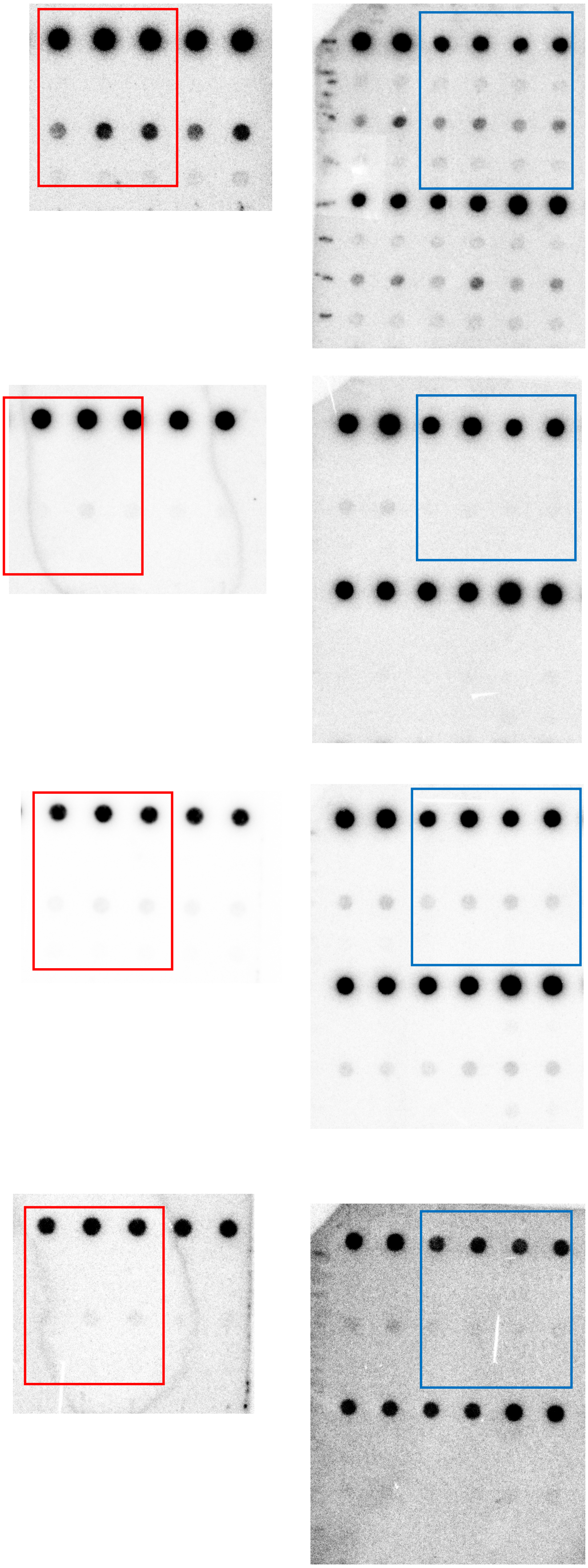

Figure 5

Figure 5a

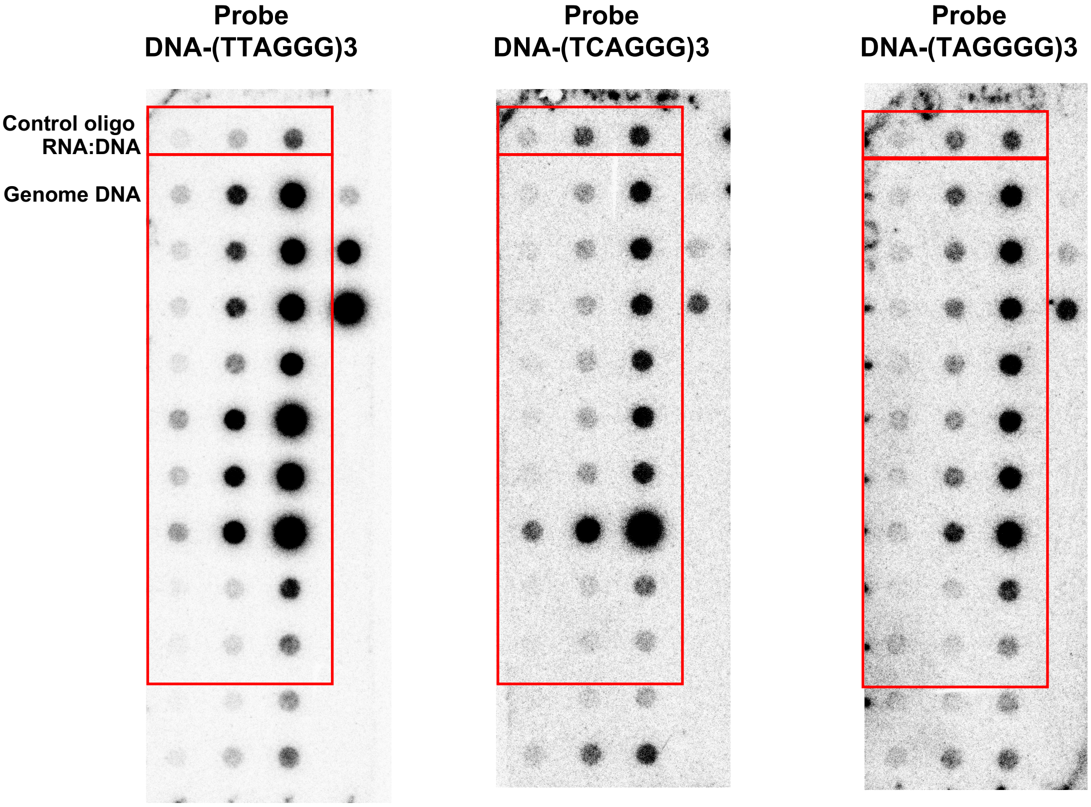

Figure 5b

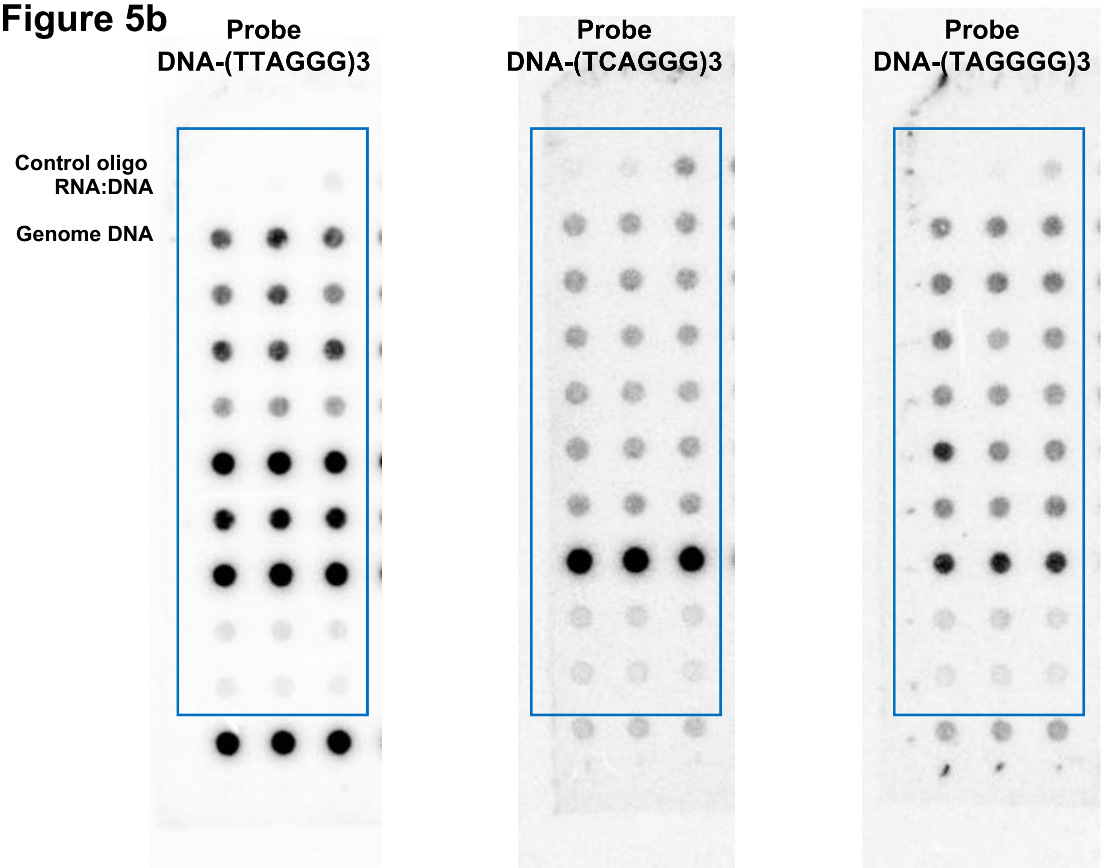

Figure 6

Figure 6c

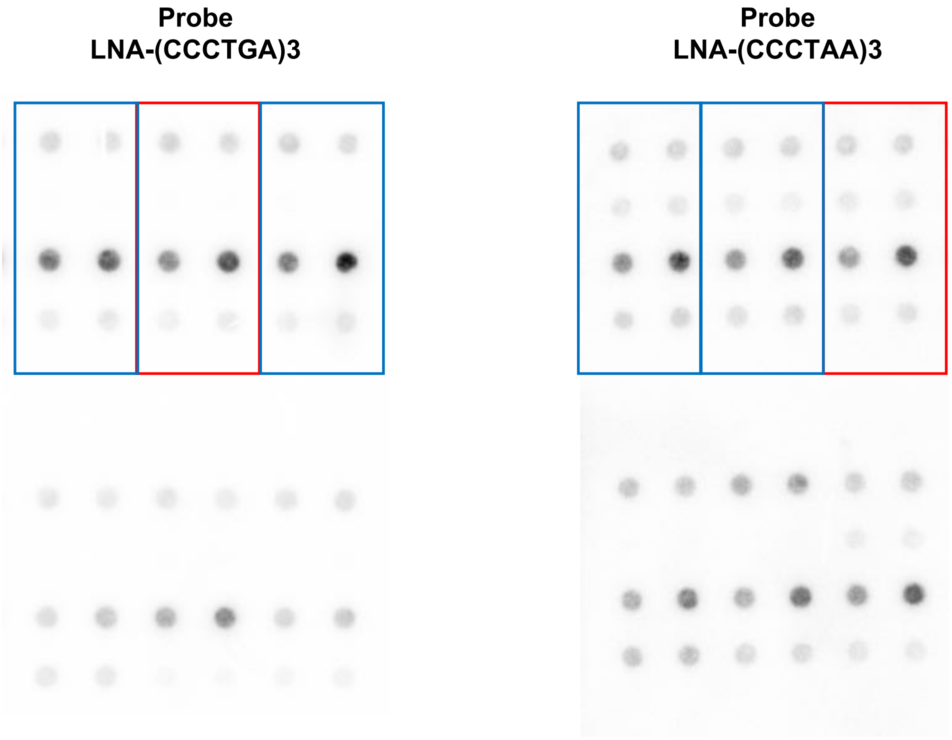

Figure 6d

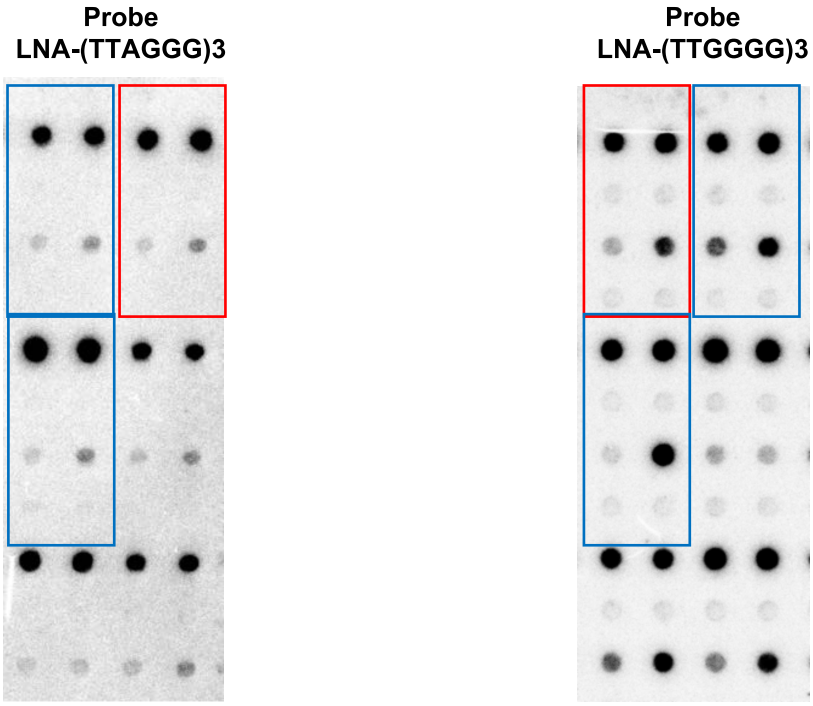

Figure 8

Figure 8a

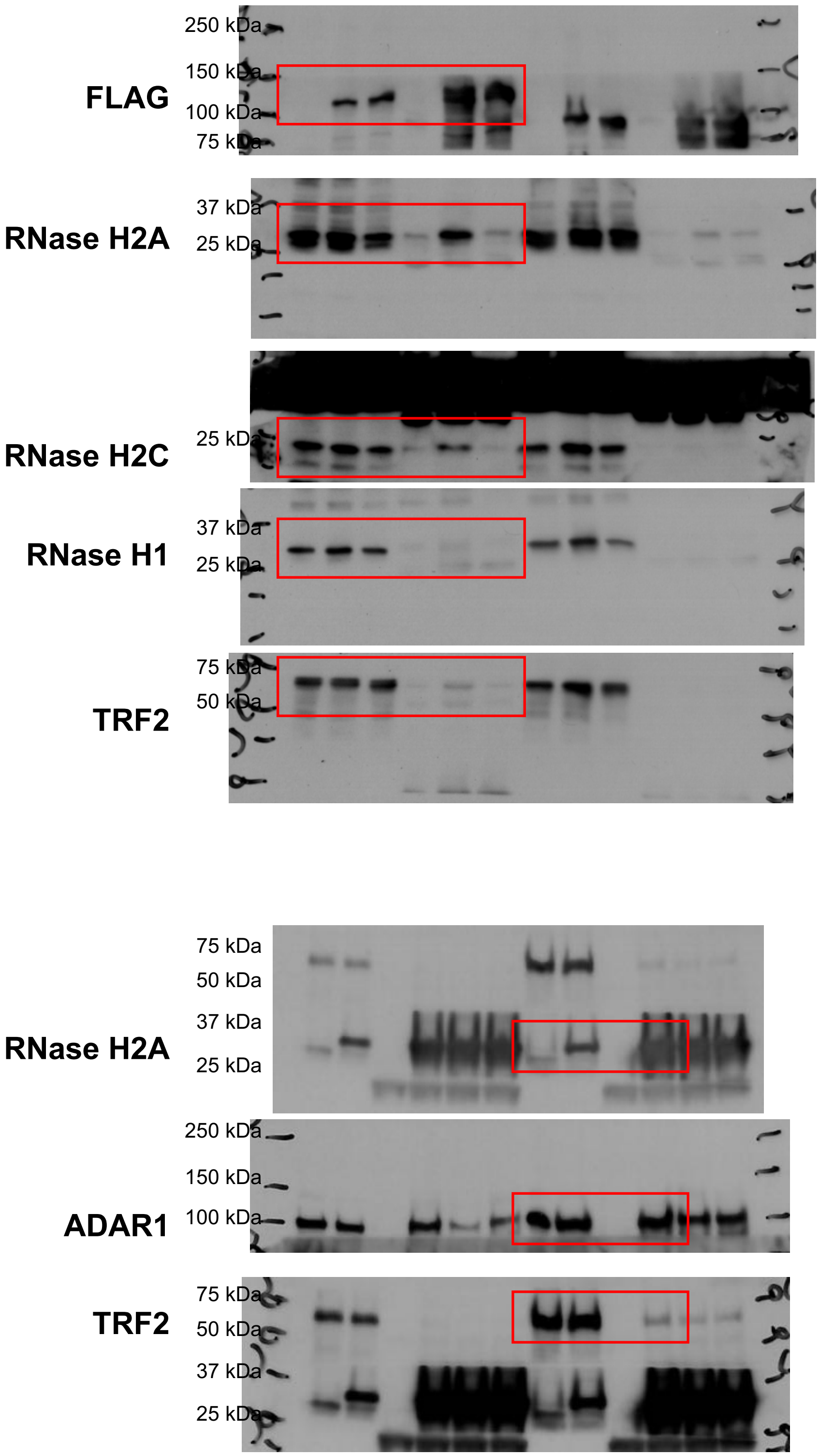

Figure 8

Figure 8b

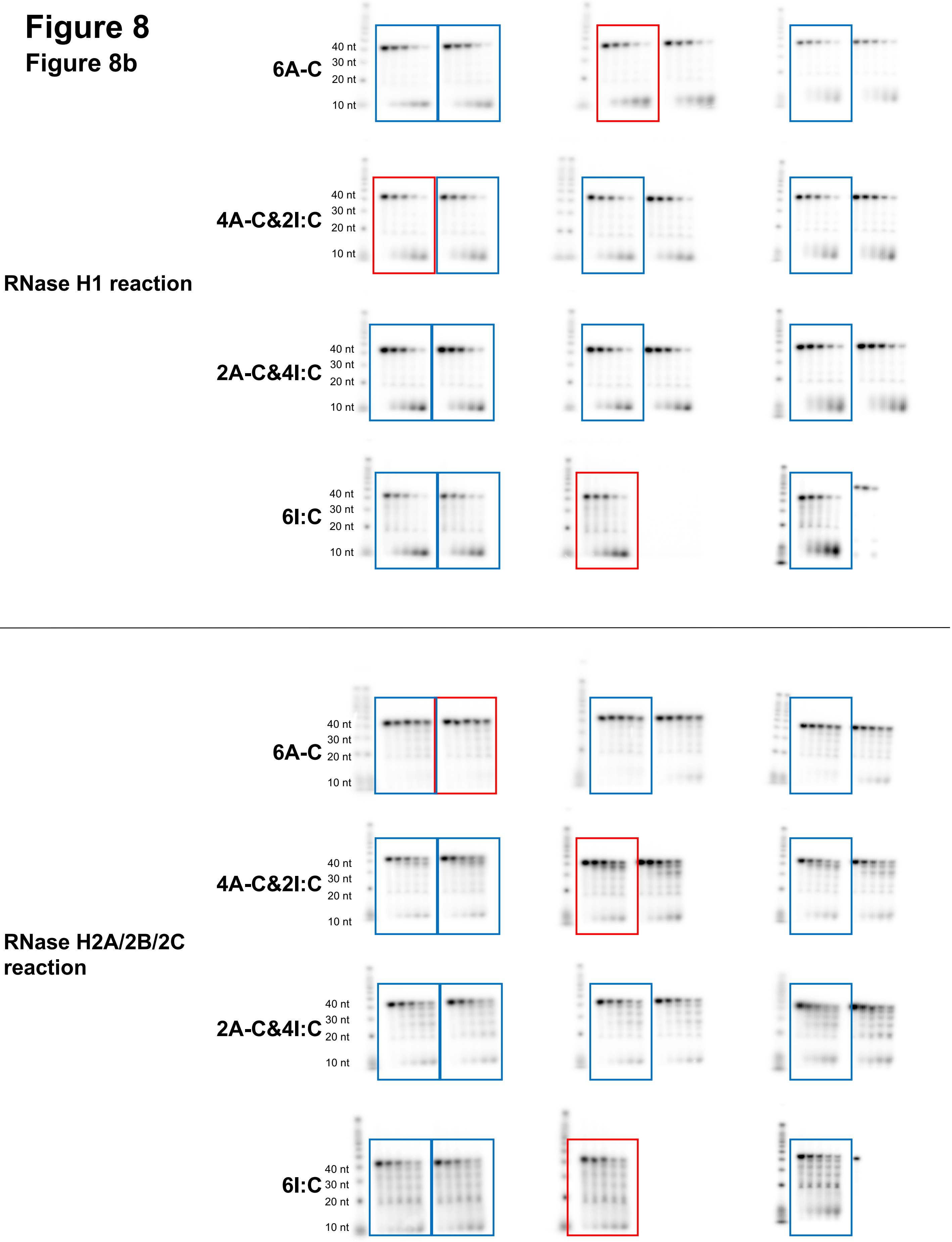

Figure 9

Figure 9a

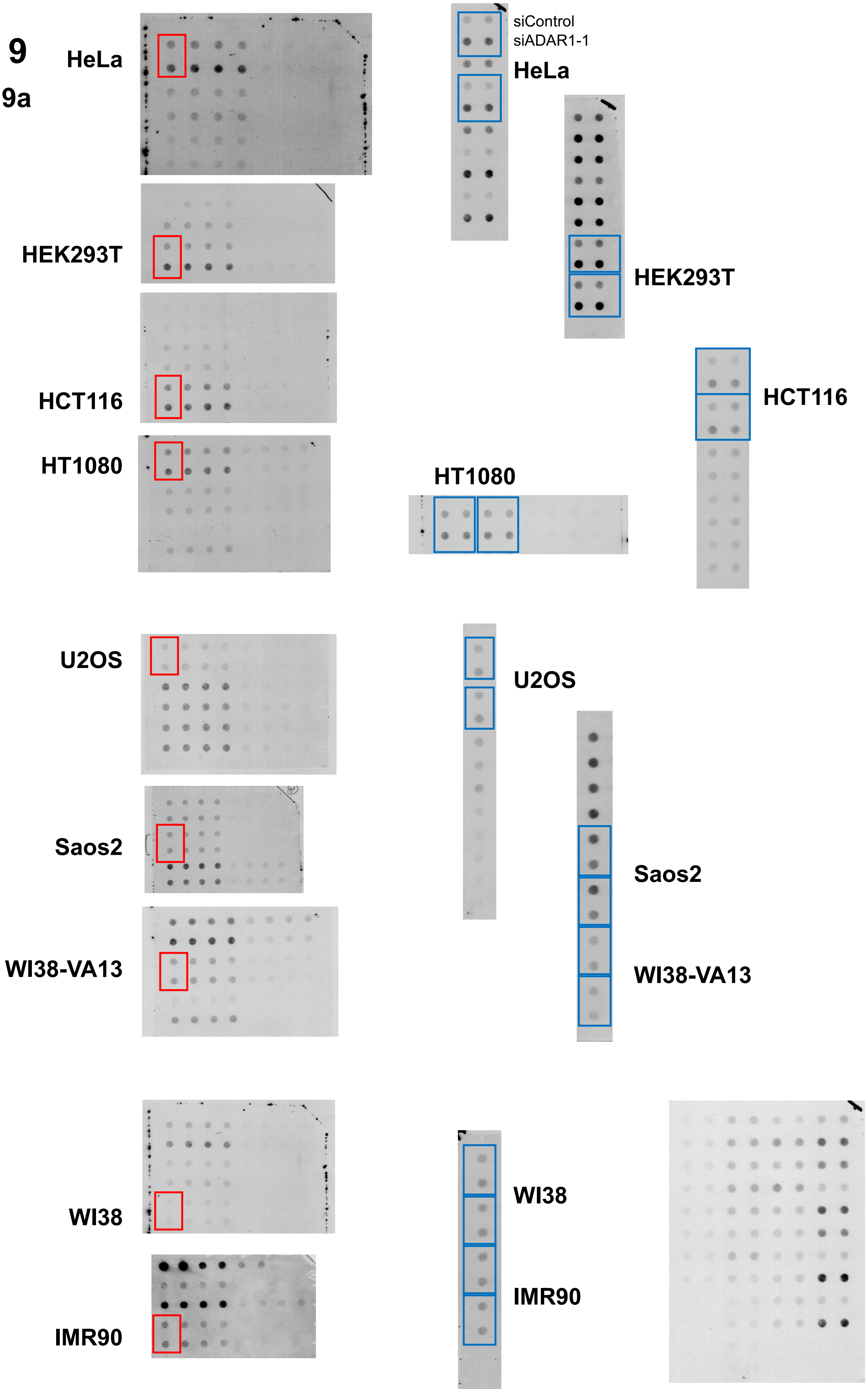

Figure 9

Figure 9b

Supplementary Figure 9

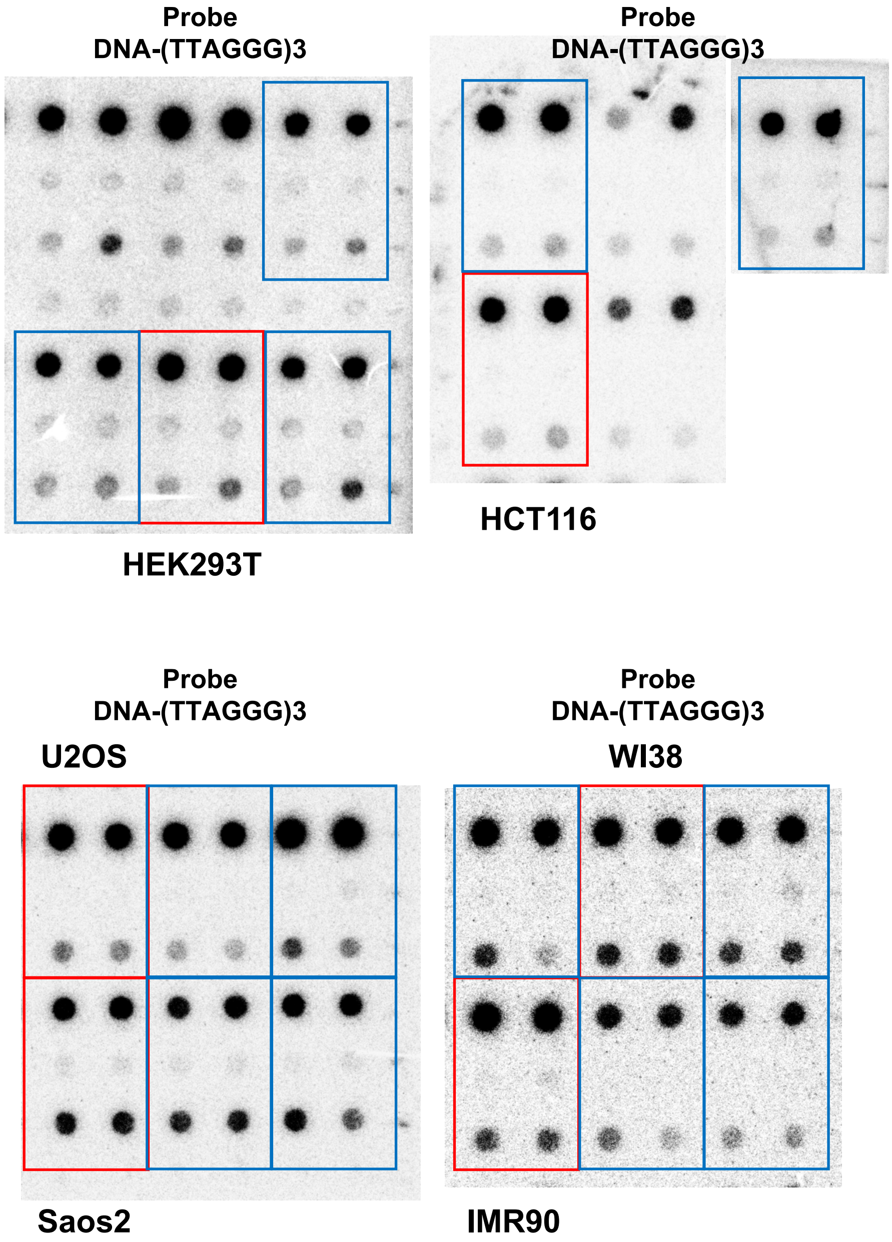

Figure 10

Figure 10a

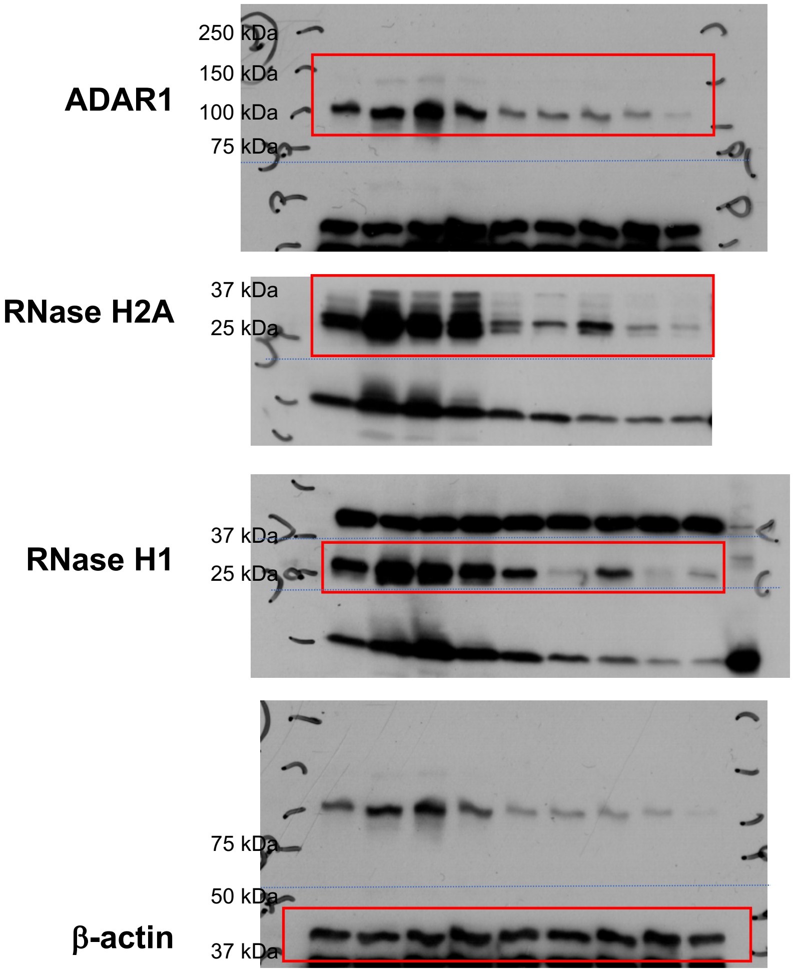

Figure 10

Figure 10b

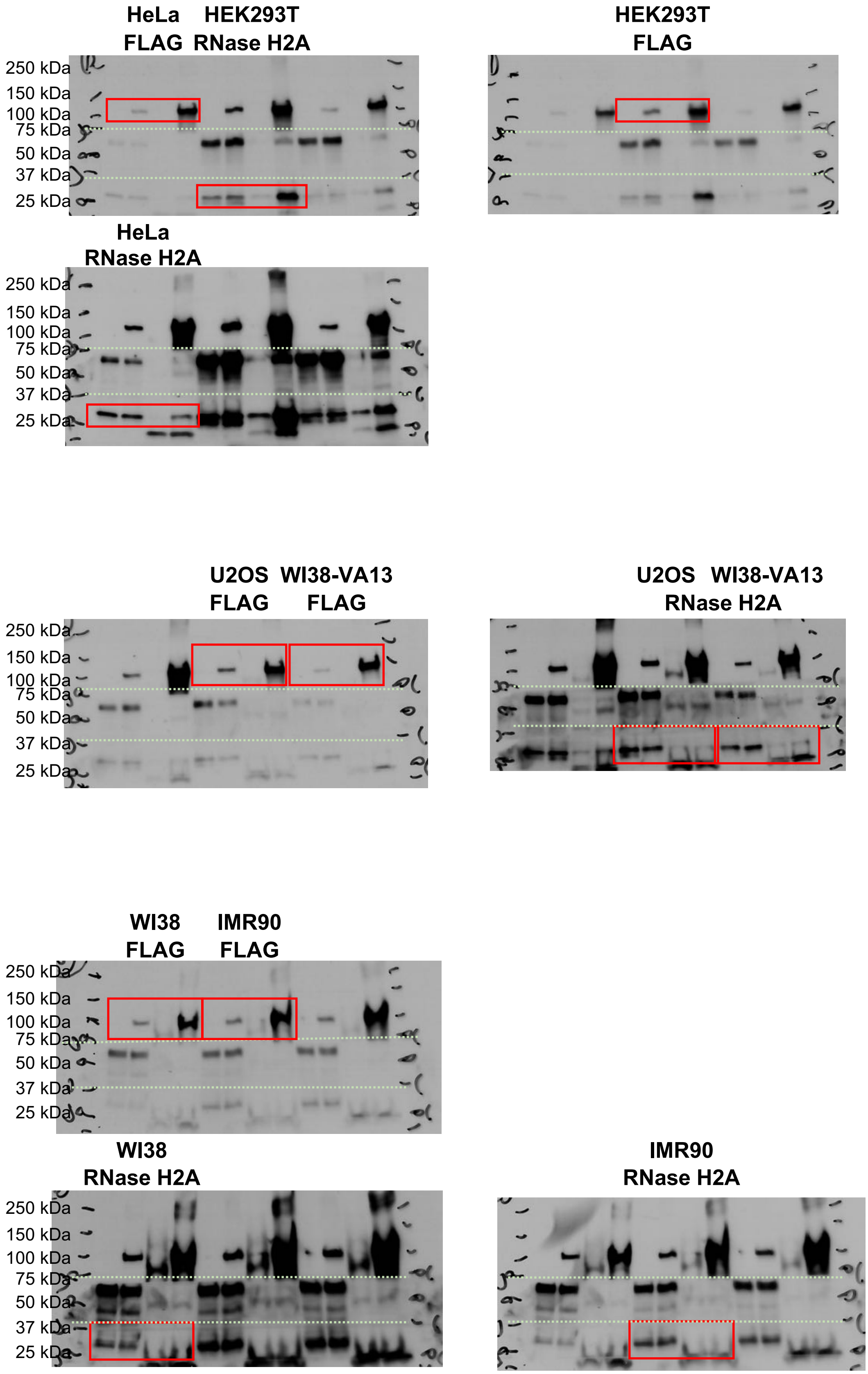

Figure 10

Figure 10c

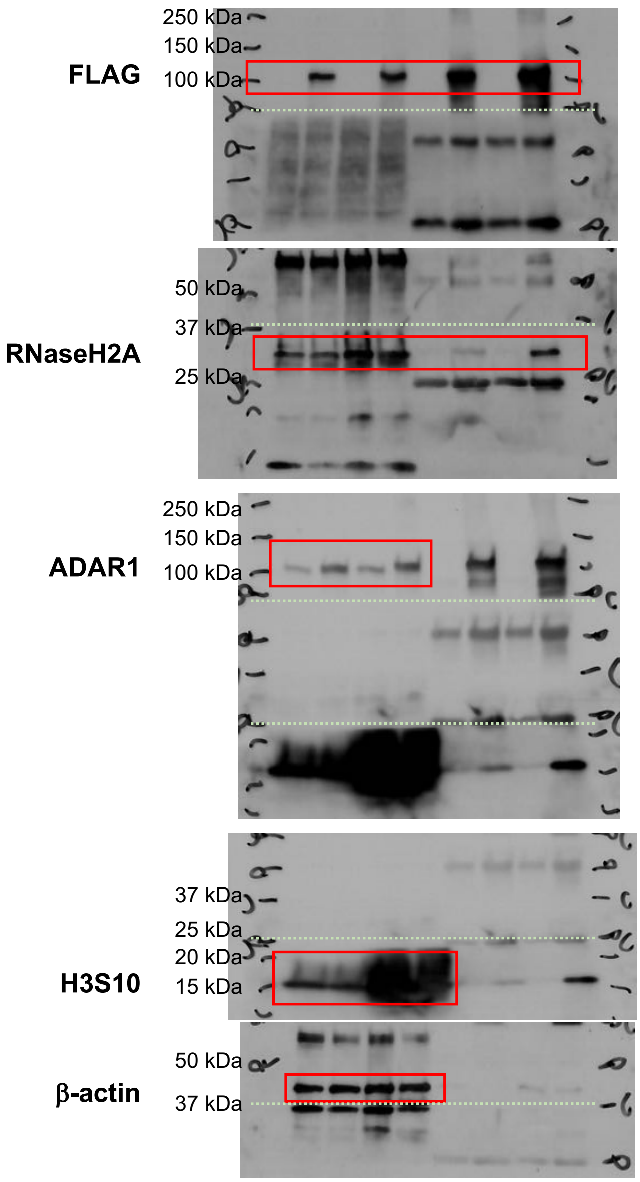

Supplementary Figure 2

Supplementary Fig. 2a

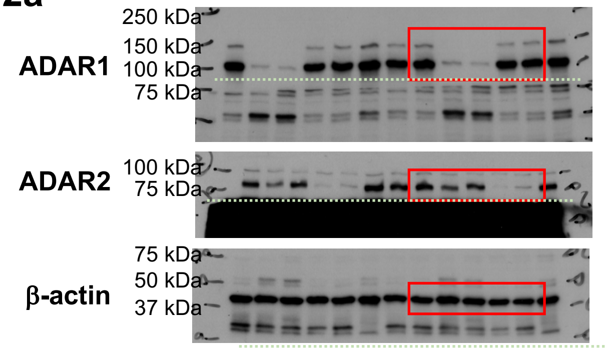

Supplementary Fig. 2b

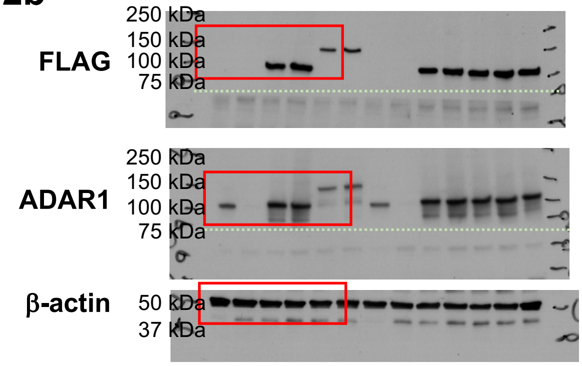

Supplementary Fig. 2c

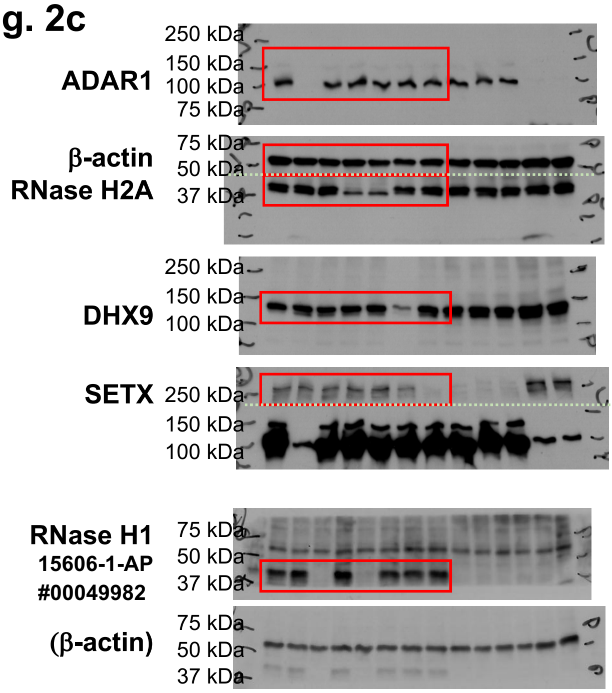

Supplementary Fig. 2d

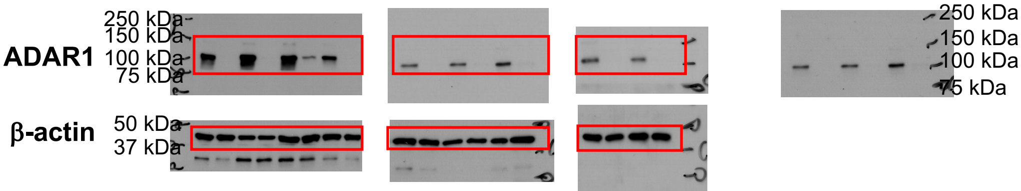

Supplementary Figure 4

Supplementary Fig. 4a

Probe DNA-(TTAGGG)3 | Probe DNA-(TCAGGG)3 | Probe DNA-(TTGGGG)3

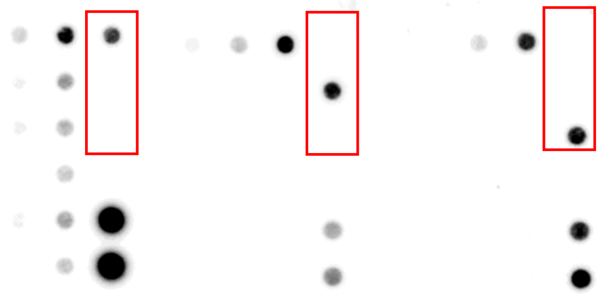

Supplementary Fig. 4b

Probe DNA-(TTAGGG)3 | Probe DNA-(TTGGGG)3

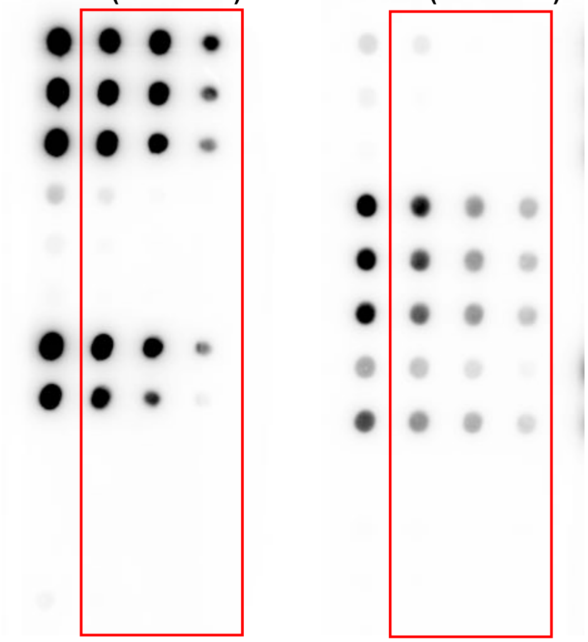

Supplementary Fig. 4c

Probe LNA-(CCCTAA)3 | Probe LNA-(CCCTGA)3

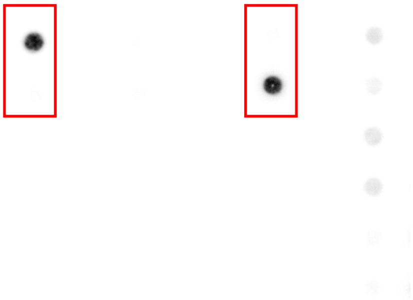

Supplementary Fig. 4d

Probe LNA-(TTAGGG)3 | Probe LNA-(TTGGGG)3

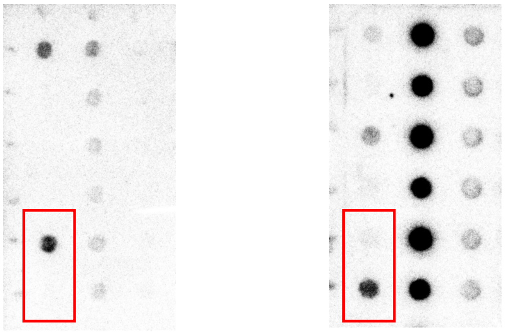

Supplementary Figure 7

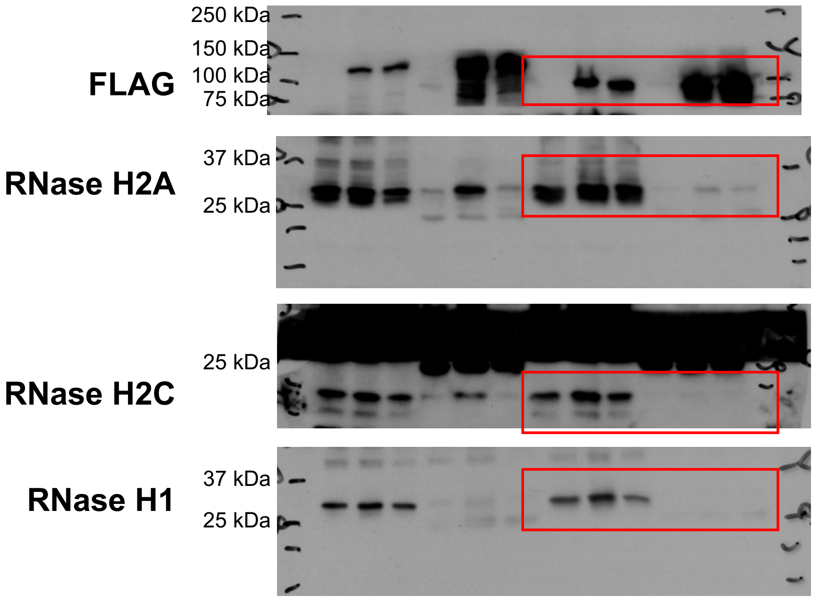

Supplement: Supplementary file 10 — Source Data [file 41467_2021_21921_MOESM10_ESM.zip › SourceDatafile2.pdf]
